# Unusual synergistic effect in layered Ruddlesden−Popper oxide enables ultrafast hydrogen evolution

**DOI:** 10.1038/s41467-018-08117-6

**Published:** 2019-01-11

**Authors:** Yinlong Zhu, Hassan A. Tahini, Zhiwei Hu, Jie Dai, Yubo Chen, Hainan Sun, Wei Zhou, Meilin Liu, Sean C. Smith, Huanting Wang, Zongping Shao

**Affiliations:** 1 Jiangsu National Synergetic Innovation Center for Advanced Materials (SICAM), State Key Laboratory of Materials-Oriented Chemical Engineering, College of Chemical Engineering, Nanjing Tech University, No. 5 Xin Mofan Road, 210009 Nanjing, P.R. China; 2 Department of Applied Mathematics, Research School of Physics and Engineering, Australian National University, Canberra, ACT 0200 Australia; 3 Max Planck Institute for Chemical Physics of Solids, Nothnitzer Strasse 40, 01187 Dresden, Germany; 4 School of Material Science and Engineering, Nanyang Technological University, 50 Nanyang Avenue, Singapore, 639798 Singapore; 5 Center for Innovative Fuel Cell and Battery Technologies, School of Materials Science and Engineering, Georgia Institute of Technology, Atlanta, GA 30332-0245 USA; 6 Department of Chemical Engineering, Monash University, Clayton, VIC 3800 Australia; 7 Department of Chemical Engineering, Curtin University, Perth, WA 6845 Australia

## Abstract

Efficient electrocatalysts for hydrogen evolution reaction are key to realize clean hydrogen production through water splitting. As an important family of functional materials, transition metal oxides are generally believed inactive towards hydrogen evolution reaction, although many of them show high activity for oxygen evolution reaction. Here we report the remarkable electrocatalytic activity for hydrogen evolution reaction of a layered metal oxide, Ruddlesden−Popper-type Sr_2_RuO_4_ with alternative perovskite layer and rock-salt SrO layer, in an alkaline solution, which is comparable to those of the best electrocatalysts ever reported. By theoretical calculations, such excellent activity is attributed mainly to an unusual synergistic effect in the layered structure, whereby the (001) SrO-terminated surface cleaved in rock-salt layer facilitates a barrier-free water dissociation while the active apical oxygen site in perovskite layer promotes favorable hydrogen adsorption and evolution. Moreover, the activity of such layered oxide can be further improved by electrochemistry-induced activation.

## Abstract (web summary)

Water may serve as a renewable hydrogen fuel source to replace fossil fuels, although such electrolysis requires highly active catalysts. Here, authors explore Ruddlesden−Popper oxides as hydrogen evolution electrocatalysts that feature an unusual synergistic effect to promote high activity.

## Introduction

With the accelerating depletion of fossil fuels and associated negative environmental impact from their excessive use, clean and renewable energy alternatives have been receiving great demand during the past years^1^. Hydrogen (H_2_), which possesses the highest gravimetric energy density and zero-carbon nature, is regarded as a promising energy carrier or fuel to replace fossil fuels^2^. Globally, hydrogen production mainly comes from the industrial steam reforming with converting natural gas and water into carbon dioxide and hydrogen, whereas the energy inefficiency of the conversion process and the existence of carbon-containing residues can lead to high costs and low purity of the hydrogen products^3^. To address this, an alternative technique to produce hydrogen is via electrochemical water splitting, which benefits from unlimited water resources, stable output, high product purity, feasibility of large-scale production, and the capability of integrating renewable energy as power source^4^. However, the rate of the hydrogen evolution reaction (HER) in water splitting is relatively sluggish and requires an efficient electrocatalyst to promote the HER process^5^. Notably, the HER kinetics for most catalysts in alkaline solutions is at least two orders of magnitude slower than in acidic ones due to the extra water dissociation step^6^. Although platinum (Pt) metal demonstrates favorable activity for HER in both acidic and alkaline media, the scarcity, high cost and poor stability severely limit the viability for commercial applications^5^. Extensive efforts have been devoted to the development of highly active and durable Pt-free HER catalysts in alkaline solutions to achieve economical hydrogen production.

As a large and important class of inorganic compounds, transition metal oxides (TMOs) have attracted great interest in many fields (e.g., efficient oxygen evolution reaction (OER) catalysts in alkaline solutions) by virtue of the merits of their stability, abundance, accessibility, and environmental friendliness^7,8^. However, bulk TMOs are generally deemed as inactive materials for alkaline HER because of poor electrical conductivity and inappropriate hydrogen adsorption energy^9,10^. For example, ruthenium dioxide (RuO_2_), a state-of-the-art OER electrocatalyst, was found to be only moderately active for HER^11,12^.

To date, several important trials have been conducted to develop oxides that can serve as efficient HER electrocatalysts. Since the mass activity of a catalyst is determined by both the intrinsic activity and the amount of active sites, increasing the amount of active sites is a direct way to increase the mass activity. Luo et al. synthesized a nonstoichiometric and mesoporous MoO_3−*x*_ with high surface area of 52 m^2^ g^−1^ via an inverse micelle-template method^13^, which shows favorable electrocatalytic activity for HER in an alkaline condition, achieving an overpotential of 138 mV at 10 mA cm^−2^. Wang et al. fabricated some TMO nanoparticles with diameters of 2–5 nm using lithium-induced electrochemical conversion reactions, demonstrating excellent activity for OER and favorable activity for HER^14^. To further increase the activity, Qiao et al. applied surface strain engineering strategy to alter the surface atom and electronic structure of nanostructured CoO, thus enhancing the alkaline HER activity^8^. Despite these efforts, the HER activity of oxides in alkaline media, especially in bulk counterpart, remains much inferior to that of the benchmark Pt catalyst; also, the synthetic processes are complex, of low yield, and difficult for cost-effective and wide-scale fabrication. Therefore, simple and effective strategies are still needed for the development of metal oxide-based electrocatalysts with high intrinsic HER activity.

In addition to the simple TMOs, complex oxides containing more than one metal (e.g., perovskite oxides) have been the focus of numerous studies for various applications by virtue of their structural and compositional flexibility^15–18^. The multiple elements and variable structures of complex oxides can give rise to some unique bulk material properties (e.g., crystal, electronic and conductive properties), which then affect the surface properties of the oxides directly or indirectly, and consequently their electrocatalytic activities^19,20^. Thus, engineering the bulk structure can provide new opportunities to screen for superior catalysts.

Here we report our findings in rational design of a layered Ruddlesden−Popper (RP)-type oxide Sr_2_RuO_4_, derived from RuO_2_, demonstrating dramatically enhanced electrocatalytic activity for HER. Although the specific surface area of bulk Sr_2_RuO_4_ is relatively low (~1.4 m^2^ g^−1^), the turnover frequency (TOF) for HER is ~10 times higher that of RuO_2_. When evaluated in 1 M KOH solution, the outstanding HER activity is reflected by an overpotential of only 61 mV at 10 mA cm^−2^ and a low Tafel slope of 51 mV dec^−1^, comparable to those of the commercial Pt/C catalyst and surpassing all metal oxides and other well-known Pt-free catalysts reported to date. Theoretical and experimental analyses reveal that an unusual synergistic effect in the layered RP structure possibly contributes to the HER activity of Sr_2_RuO_4_: SrO-terminated surface cleaved in rock-salt layer promotes water dissociation without a barrier while the active apical oxygen site in the perovskite layer enables favorable hydrogen adsorption and evolution. Besides, the HER activity of Sr_2_RuO_4_ can be further improved by the interface synergy between in situ formation of Ru nanoparticle and oxide substrate over the surface as well as incremental electrochemical surface area (ECSA) during electrochemistry-induced activation process. The design strategy of the layered RP Sr_2_RuO_4_ oxide and the derived synergistic effect for HER catalysis may be applicable to knowledge-based design of more efficient nonprecious electrocatalysts.

## Results

### Bulk structure characterization

**Fig. 1:**
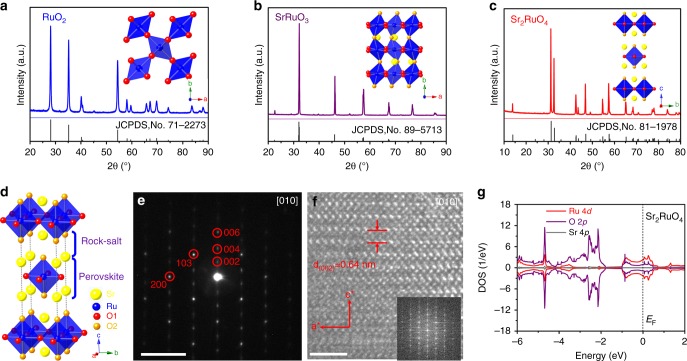
Bulk structure characterization. XRD patterns of **a** RuO_2_, **b** SrRuO_3_, and **c** Sr_2_RuO_4_. Insert shows the local crystal structure of respective oxide. Color code: Sr (yellow), Ru (blue), and O (red and orange). **d** Schematic presentation of the layered RP structure of Sr_2_RuO_4_. **e** SAED pattern and the corresponding HRTEM images (**f**) along the [010] zone axes for Sr_2_RuO_4_. Insert is the FFT. **g** DFT calculation of the DOS of Sr_2_RuO_4_. Scale bar in (**e**) is 5 nm^−1^ and in (**f**) is 2 nm

Since RuO_2_ has only modest activity for HER in the alkaline solution, the electrocatalytic activity of several Ru-based oxides with different crystal structures was characterized to determine the effect of crystal structure on activity. Figure 1a–c shows the X-ray diffraction (XRD) patterns of RuO_2_, SrRuO_3_, and Sr_2_RuO_4_ powders synthesized using a solid-state reaction process. All diffraction peaks of the three oxide samples match well with those of the corresponding standard patterns (JCPDS No. 71-2273, 89-5713, and 81-1978, respectively), indicating their phase purity without observable impurities. This is also confirmed by the Rietveld refinement of the XRD patterns (Supplementary Fig. 1); the space group is *P*42*/mnm* for RuO_2_, *Pnma* for SrRuO_3_, and *I*4/*mmm* for Sr_2_RuO_4_. The local crystal structure and overall network of the [RuO_6_] octahedra in the three structures are schematically shown in inserted Fig. 1a–c based on the Rietveld analysis along with the detailed structure parameters (Supplementary Table 1). Particularly, Sr_2_RuO_4_ oxide crystallizes in the K_2_NiF_4_-type structure with layers of corner-linked [RuO_6_] octahedra separated by [SrO] rock-salt layers along the *c*-direction, which is schematically illustrated in Fig. 1d. Actually, Sr_2_RuO_4_ belongs to the so-called RP series with the formula Sr_*n*+1_Ru_*n*_O_3*n*+1_^21^. These strontium ruthenates consist of alternative perovskite SrRuO_3_ layer and SrO rock-salt layer. The layered RP crystal structure of Sr_2_RuO_4_ is further confirmed by the selected-area electron-diffraction (SAED) pattern along the [010] zone axes and the corresponding high-resolution transmission electron microscopy (HRTEM) image. The SAED pattern in Fig. 1e and fast Fourier transform (FFT) images insert in Fig. 1f clearly reflect the tetragonally arranged diffraction spots of [010] zone axes. In addition, a lattice fringe with lattice spacing of 0.64 nm was observed in the HRTEM image (Fig. 1f), corresponding to the (002) plane of Sr_2_RuO_4_ oxide. X-ray absorption spectroscopy (XAS) technique was further performed to analyze the oxidation state of Ru in the Sr_2_RuO_4_. Supplementary Fig. 2 shows the Ru-*L*_2,3_ XAS spectra of the as-synthesized Sr_2_RuO_4_ sample and the reference samples with different valence states of Ru. The Ru-*L*_2,3_ spectra of the as-synthesized Sr_2_RuO_4_ sample is completely the same as the reference Sr_2_RuO_4_ (including spectra shape and peak position), suggesting the character of stoichiometry and only Ru^4+^ in the as-synthesized Sr_2_RuO_4_ sample.

The morphologies of RuO_2_, SrRuO_3_, and Sr_2_RuO_4_ powders were examined by scanning electron microscopy (SEM) as displayed in Supplementary Fig. 3. Micrometer-sized particles were observed, suggesting the bulk nature of the as-synthesized SrRuO_3_ and Sr_2_RuO_4_ oxides by solid-state reaction method. The RuO_2_ appears to have smaller particle size than SrRuO_3_ and Sr_2_RuO_4_, and accordingly have larger surface areas as calculated from the Brunauer−Emmett−Teller (BET) measurements (Supplementary Fig. 4).

We also use density functional theory (DFT) calculations to unveil the electronic structures of RuO_2_, SrRuO_3_, and Sr_2_RuO_4_ by calculating the density of states (DOS) (Fig. 1g and Supplementary Fig. 5). It can be found clearly that the DOS profiles of Ru and O change significantly among the three oxides, accordingly having different electronic structures. Nevertheless, the three oxides all present a typical metallic behavior with predominantly Ru 4*d* and O 2*p* orbital crossing the Fermi level, which is highly beneficial to electrocatalysis due to high electronic conductivity^10^. Based on the above combined analysis, the Sr_2_RuO_4_ exhibits distinctive structural features (e.g., alternative layers, [RuO_6_] coordination geometry, Ru−O bond length, states near Fermi level) relative to RuO_2_ and SrRuO_3_, although they have the same oxidation state of Ru^4+^ and similar morphology. These structural features in Sr_2_RuO_4_ are the key factors for enhancing the HER activity, as to be discussed below.

### Comprehensive evaluation of HER activity

**Fig. 2:**
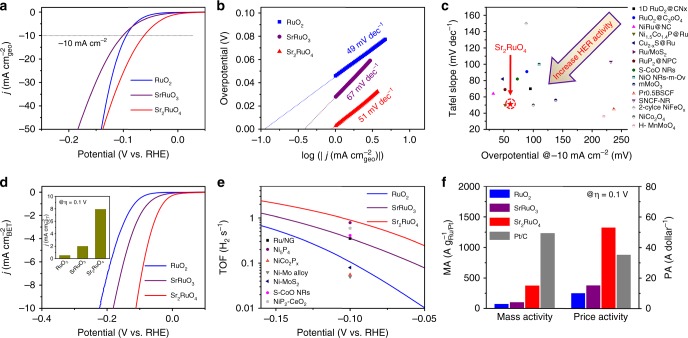
Electrocatalytic HER activity. **a** Polarization curves and corresponding **b** Tafel plots of RuO_2_, SrRuO_3_, and Sr_2_RuO_4_ catalysts in an Ar-saturated 1 M KOH solution. Scan rate, 5 mV s^−1^. **c** Alkaline HER activity comparison graph showing the Tafel slope with overpotential@−10 mA cm^−2^. **d** Specific activity normalized to BET surface area of RuO_2_, SrRuO_3_, and Sr_2_RuO_4_ catalysts as a function of potential. Inset: specific activity at the overpotential of *η* = 0.1 V. **e** The relationship between TOF and the tested potentials for RuO_2_, SrRuO_3_, and Sr_2_RuO_4_ electrocatalysts in 1 M KOH solution. TOF values of some well-known HER catalysts in the literature are also shown for comparison. **f** Mass activity (MA) and price activity (PA) of RuO_2_, SrRuO_3_, Sr_2_RuO_4_ and Pt/C at the overpotential of *η* = 0.1 V

To evaluate the electrocatalytic HER activity of RuO_2_, SrRuO_3_, and Sr_2_RuO_4_ oxides with different crystal structures in alkaline media, an electrochemical cell with a three-electrode configuration in an Ar-saturated 1 M KOH solution was used. Unless indicated otherwise, all potentials in this study were referenced to a reversible hydrogen electrode (RHE, see Supplementary Fig. 6 for RHE calibration) and *iR*-corrected to eliminate the ohmic potential drop across the electrolyte. Figure 2a shows the polarization curves gained from linear sweep voltammetry at a scan rate of 5 mV s^−1^. Sr_2_RuO_4_ can effectively catalyze alkaline HER with an extremely low onset overpotential (defined here as the overpotential at 1 mA cm^−2^) of ~3 mV; as a comparison, RuO_2_ and SrRuO_3_ show much larger onset potential of ~46 and ~28 mV, respectively. It is also seen that the added conductive carbon, which is always identified as a conductive additive and support in oxide electrodes^22–24^, displays negligible HER activity (Supplementary Fig. 7). Besides, the Sr_2_RuO_4_ requires a small overpotential of 61 mV to obtain a current density of −10 mA cm^−2^, much lower than that of the RuO_2_ (95 mV) and SrRuO_3_ (101 mV) catalysts. The result suggests that the [RuO_6_] octahedra layer in the Sr_2_RuO_4_ structure has much higher electrocatalytic activity for HER than those in the other two crystal structures.

To gain more insight into the electrochemical behavior of the electrocatalysts, polarizations in a broader range of overpotential were acquired and presented in Fig. 2b. In general, HER in an alkaline electrolyte includes two steps, i.e., electron-coupled water dissociation (the formation of H_ad_ by the Volmer step) and the concomitant transformation of H_ad_ into molecular H_2_ (the Heyrovsky or Tafel step)^5,25^. A Tafel slope for the Volmer, Heyrovsky, and Tafel step, as the rate-determining step for HER, is expected to be ~120, ~40, and ~30 mV dec^−1^ respectively^5,25^. Sr_2_RuO_4_ yields a Tafel slope of 51 mV dec^−1^, which is close to the RuO_2_ (49 mV dec^−1^) and SrRuO_3_ (67 mV dec^−1^), suggesting hydrogen evolution via Volmer−Heyrovsky mechanism in the alkaline electrolyte for all three oxides, and that the Heyrovsky step is the rate-limiting step. Also, small Tafel slope of Sr_2_RuO_4_ implies fast HER kinetics. Furthermore, the exchange current density (*j*_0_) was obtained from extrapolating the Tafel plots to zero overpotential. The *j*_0_ is dependent sensitively on the nature of the catalyst material and determines the rate of the intrinsic electron transfer between the catalyst and the electrolyte solution^26^. The *j*_0_ value of Sr_2_RuO_4_ is 0.898 mA cm^−2^, which is ~8-fold and ~2-fold higher than that of RuO_2_ (0.115 mA cm^−2^) and SrRuO_3_ (0.409 mA cm^−2^), respectively. As a whole, the high HER kinetic metrics (including the small overpotential, low Tafel slope, and high exchange current density) highlight the outstanding catalytic activity of Sr_2_RuO_4_ for HER in alkaline media, in particular by considering its small specific surface area (1.4 m^2^ g^−1^). Such excellent HER activity of Sr_2_RuO_4_ is superior to those of other metal oxides and Ru-based catalysts (Fig. 2c) as well as various well-known active Pt-free catalysts reported to date. A detailed HER activity comparison with previously reported representative HER catalysts, including metal/alloy, metal phosphides, metal sulfides, metal selenides, metal carbides, functional carbon and composites etc., demonstrates that Sr_2_RuO_4_ is among the most active HER catalyst in alkaline media (Supplementary Table 2).

It is well known that the overall catalytic activity of an electrocatalyst system is generally determined by two key factors: the intrinsic activity of each active site and the number of active sites^27^. To assess the intrinsic activity of RuO_2_, SrRuO_3_, and Sr_2_RuO_4_ oxides, it is necessary to eliminate the contribution derived from the surface areas and thereby calculate the specific activity (SA) normalized to the real oxide surface area or ECSA. The real oxide surface area and ECSA are estimated from the BET measurements (Supplementary Fig. 4) and double-layer capacitance (*C*_dl_) measurements (Supplementary Fig. 8), respectively. The *C*_dl_ obtained from a cyclic voltammetry (CV) method is expected to be linearly proportional to the ECSA^5,25^. As shown in Fig. 2d and Supplementary Fig. 9, the SA of Sr_2_RuO_4_ is much higher than that of RuO_2_ and SrRuO_3_, regardless of the current densities normalized by the BET surface area or ECSA. For example, at *η* = 0.1 V, the Sr_2_RuO_4_ delivers an SA of 7.93 mA cm^−2^_BET_, which is ~15 and ~4 times higher than that for RuO_2_ and SrRuO_3_. The TOF, which is associated with the number of H_2_ molecules evolved per second per active site, is another important parameter that characterizes the intrinsic activity of an electrocatalyst^28–30^. In this work, TOF values were calculated according to a well-known method reported by the Jaramillo group (see the Supplementary Information for detailed calculations, Supplementary Fig. 10 and Supplementary Note 1)^29,30^. Figure 2e shows the TOF (per surface active O atom) versus potential plots of RuO_2_, SrRuO_3_, and Sr_2_RuO_4_ catalysts, and it reveals that the TOF values follows the order in the sequence of Sr_2_RuO_4_ > SrRuO_3_ > RuO_2_. Particularly, the Sr_2_RuO_4_ electrocatalyst delivers an extremely high TOF value of 0.90 s^−1^ at overpotential (*η*) of 0.1 V, which is evidently larger than that of some well-known HER catalysts in 1 M KOH solution, e.g., Ru/NG (0.35 s^−1^), Ni_5_P_4_ (0.79 s^−1^), NiCo_2_P_*x*_ (0.056 s^−1^), Ni-Mo alloy (0.05 s^−1^), Ni-MoS_2_ (0.08 s^−1^), S-CoO NRs (0.41 s^−1^), NiP_2_-CeO_2_ (0.593 s^−1^) (Supplementary Table 3). According to the HER activities of RuO_2_, SrRuO_3_, and Sr_2_RuO_4_ summarized in Supplementary Table 4, the intrinsic HER activity of the layered RP Sr_2_RuO_4_ oxide is almost one order of magnitude higher than that of the RuO_2_, yet the ruthenium content is much less, only ~61 wt% of that in RuO_2_, producing the dual beneficial effects of activity enhancement and cost reduction simultaneously. The HER activity was further confirmed by the Faradaic efficiency, which is assessed by comparing the theoretical and detected volumes of generated gases during potentiostatic electrolysis experiments. As shown in Supplementary Fig. 11, the detected volume of gases matches the theoretical one, implying a high Faraday efficiency close to 100%. Moreover, the Sr_2_RuO_4_ oxide catalyst prepared by a simple solid-state reaction method enables HER activity comparable to that of the commercial Pt/C catalyst (Supplementary Fig. 12 and Supplementary Fig. 13). Although Sr_2_RuO_4_ still exhibits somewhat lower mass activity than the commercial Pt/C, it is more advantageous in price activity (Fig. 2f) and SA (Supplementary Fig. 14) considering the difference in their price (Supplementary Table 5) and specific surface area. It is also worthwhile to note that Sr_2_RuO_4_ shows much better durability than the benchmark Pt/C catalyst, demonstrating its value for practical application (Supplementary Fig. 15). The poor durability of Pt/C catalyst may be related to the dissolution of Pt surface atoms, the agglomeration of Pt particles, and the corrosion of the carbon support^31,32^.

### Theoretical study

**Fig. 3:**
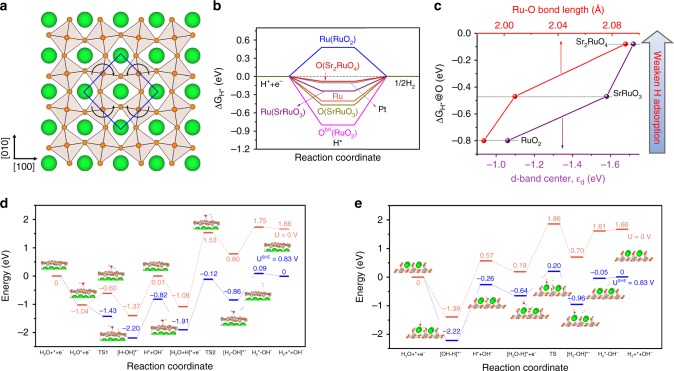
DFT calculations. **a** Top view of the SrO-terminated Sr_2_RuO_4_ (001) surface. The RuO_6_ octahedra are alternately tilted with respect to the *c-*axis, and rotated in the *ab* plane as indicated by the curved arrows. **b** Free-energy diagram for hydrogen adsorption at the metal and oxygen sites on Sr_2_RuO_4_, SrRuO_3_, RuO_2_, Ru, and Pt. **c** Relationship between Δ*G*_H*_@O and d-band center as well as Ru−O bond length. Energy diagram and the simplified surface structures of the various reaction species along the Volmer−Heyrovsky pathway for HER in alkaline media on the **d** RuO_2_-terminated surface of SrRuO_3_ and **e** SrO-terminated surface of Sr_2_RuO_4_ at electrode potential *U* = 0 V and *U*^SHE^ = 0.83 V, respectively. TS transition state. The gray, green, orange/red, and light gray spheres represent Ru, Sr, O, and H atoms, respectively

Clearly, the layered RP Sr_2_RuO_4_ oxide exhibits significantly higher HER activity than the simple oxide RuO_2_ and perovskite oxide SrRuO_3_ in alkaline media. To explore the origin of the high electrocatalytic activity, we resorted to DFT calculations. HER in an alkaline solution that is considered to proceed via two steps^5,8,9,25,28^, i.e., either Volmer−Heyrovsky or Volmer−Tafel pathways (Volmer: * + H_2_O + e^−^ → H* + OH^−^; Heyrovsky: H* + H_2_O + e^−^ → H_2_ + OH^−^; Tafel: H* + H* → H_2_, where * is the active site). Based on the above analysis of Tafel slope, the three oxides including Sr_2_RuO_4_ underwent a Volmer−Heyrovsky pathway in alkaline media. TMOs are generally regarded as unfavorable catalysts for converting H* to H_2_ (either too strong H* adsorption on oxygen ion or too weak H* adsorption on metal ion)^8,9,29,33^; thus we first studied hydrogen adsorption on the catalysts surface. The adsorption-free energy of H* (Δ*G*_H*_) is a key descriptor of the HER activity in both alkaline and acidic conditions, and generally a Δ*G*_H*_ value close to zero results in superior HER activity due to the optimal balance between absorption and desorption of hydrogen atoms on the active sites^9,10,28,34,35^. Hence, the Δ*G*_H*_ at the metal site or oxygen (O) site for RuO_2_, SrRuO_3_, and Sr_2_RuO_4_ was calculated using DFT according to the as-built catalyst structural models (Supplementary Fig. 16-18), along with Ru and Pt metals as references. Notably, the (001) SrO-terminated surface of Sr_2_RuO_4_, the (010) RuO_2_-terminated surface of SrRuO_3_, (110) surface of RuO_2_, and (0001) surface of Ru and (111) surface of Pt were used in our theoretical investigation considering that they are among the most usually observed and most stable ones in both experimental and theoretical studies^21,28,36–39^. Besides, based on previous findings that octahedral rotation happens on the surface of Sr_2_RuO_4_^21,40^, the RuO_6_ octahedral unit rotated alternatingly by ~8.4° is also considered (Fig. 3a). Figure 3b shows the Δ*G*_H*_ values on all the possible sites for the studied samples. The Δ*G*_H*_ value on Ru and O site for RuO_2_ is 0.48 and −0.8 eV, respectively, indicating that the H* adsorption is either too weak or too strong. Consequently, the HER kinetics are sluggish on the RuO_2_ surface. For SrRuO_3_, the H binding strength is relatively too strong regardless of whether H adsorbs on Ru or O site, resulting in suppressed H desorption and H_2_ production is thus hindered. Impressively, the Δ*G*_H*_ value on the O site of Sr_2_RuO_4_ is only −0.08 eV, which is most close to ideal zero and very similar to Pt (−0.10 eV), suggesting an optimum hydrogen adsorption strength on the surface of Sr_2_RuO_4_. The H* adsorption ability of transition metal compounds at the nonmetal site has been recently reported to relate to the electronic structure of metal elements (e.g., d-band center)^41,42^ and geometry factors (e.g., bond length)^41,43,44^. Thus, the d-band center of Ru (Supplementary Fig. 19) and Ru−O bond length were calculated for RuO_2_, SrRuO_3_, and Sr_2_RuO_4_ oxides. As seen from Fig. 3c, the Δ*G*_H*_@O value increases as the d-band center downshifts and Ru−O bond length increases, which is consistent with previous resports^41^. Especially, Sr_2_RuO_4_ delivers weaker H adsorption ability resulting from the downshift of the d-band center and the increase of bond length compared to SrRuO_3_ and RuO_2_, generating optimal level (close to 0) contributing to the highest HER activity. It is worth noting that there are only small variations in the adsorption energies calculated in vacuum and with solvation effects considered (Supplementary Table 6). In addition, the coverage effect of H on Δ*G*_H*_ for Sr_2_RuO_4_ and SrRuO_3_ was also considered. The results as shown in Supplementary Fig. 20 support the conclusion that Sr_2_RuO_4_ can achieve higher coverages with lower potentials while the same is not necessarily the case for SrRuO_3_ as going from low coverage to high coverage involves high potentials (*θ*_H_ = 0.25–0.5 ML).

Notably, apart from the Δ*G*_H*_ value, the water dissociation kinetics is also very crucial to the overall reaction rate for the alkaline HER^4,8,9,28,35^, which can be reflected by the activity difference between acidic and alkaline media of Pt/C and Sr_2_RuO_4_ catalysts. For benchmark Pt/C catalyst, the HER activity in an alkaline solution is lower than that in an acidic solution (Supplementary Fig. 21), indicating sluggish water dissociation in the catalytic process. Nevertheless, judged from the overpotential and TOF, Sr_2_RuO_4_ exhibits higher HER activity in an alkaline solution than in an acidic solution (Supplementary Fig. 22), due likely to faster kinetics for water dissociation, as demonstrated by Diebold et al. using low-temperature scanning tunneling microscopy (STM)^21^. They found that this layered RP structure easily cleaves apart in the rock-salt layer, and water dissociation is facile on the SrO-terminated surface^21^. We further performed DFT calculation of the energies for the water dissociation to demonstrate this phenomenon. To gain a more comprehensive picture about the H_2_ evolution reaction mechanism, we modeled the complete Volmer−Heyrovsky mechanism occurring at the most stable surfaces of Sr_2_RuO_4_ and SrRuO_3_. As displayed in Fig. 3d, e, Sr_2_RuO_4_ shows much lower energy barrier for water dissociation as compared with SrRuO_3_. Strikingly, on the SrO-terminated surface of Sr_2_RuO_4_, the kinetics to split water molecules is very favorable with the first water molecule splitting via a barrierless pathway. For the second water molecule splitting via the Heyrovsky step (H* + H_2_O + e^−^ → H_2_ + * + OH^−^), the energy barrier is 1.66 eV at *U* = 0 V and can drop to 0.84 eV at *U*^SHE^ = 0.83 V. In contrast, SrRuO_3_ presents large barriers for water dissociation on the most stable RuO_2_-termination surface, in particular for the second water molecule in the Heyrovsky step where the barrier is about 2.6 eV at *U* = 0 V. The facile water dissociation results in strong hydroxylation on the surface of Sr_2_RuO_4_, which was characterized by X-ray photoelectron spectroscopy (XPS). XPS spectra of O 1s species (Supplementary Fig. 23) and the corresponding deconvolution results (Supplementary Table 7) demonstrate much larger number of −OH on the surface for Sr_2_RuO_4_. Abundant −OH on the surface was further confirmed by considering the Sr 3d spectra of Sr_2_RuO_4_ (Supplementary Fig. 24), and the result shows the formation of hydroxide surface species. Previous studies have proven that (hydro-)oxides can effectively promote the dissociation of water^9,33,45,46^, and thereby it is believed that abundant −OH specie on the surface of Sr_2_RuO_4_ is beneficial additionally to the water dissociation. Therefore, by theoretical calculations, we reasonably identified the origin of perfect HER performance of the layered RP Sr_2_RuO_4_ catalyst. Briefly, the outstanding HER catalytic activity in alkaline media of Sr_2_RuO_4_ oxide may be ascribed to an unusual synergistic effect in the RP layered structure, whereby the SrO-terminated surface cleaved in the rock-salt layer promotes water dissociation and the apical oxygen site in the perovskite layer enables favorable hydrogen adsorption for recombination into H_2_.

### Further activity improvement induced by electrochemical activation

**Fig. 4:**
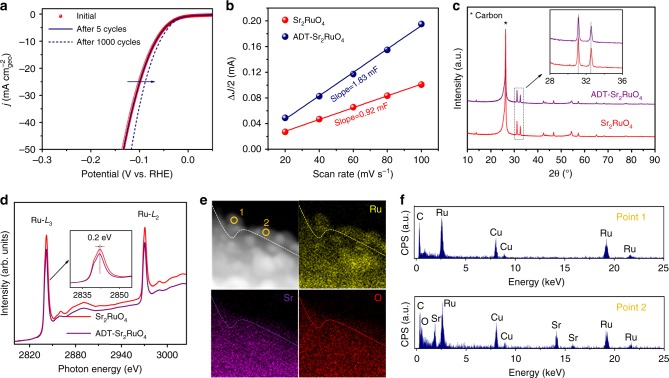
Electrochemical activation of Sr_2_RuO_4_ by ADT. **a** Polarization curves of Sr_2_RuO_4_ catalyst initially, as well as after 5 and 1000 cycles. **b** Linear fitting curves of the capacitive currents versus CV scan rates for Sr_2_RuO_4_ and ADT-Sr_2_RuO_4_. **c** XRD patterns of Sr_2_RuO_4_ and ADT-Sr_2_RuO_4_ loaded on the carbon paper substrate. Insert is the magnified XRD patterns showing main peaks of Sr_2_RuO_4_ and ADT-Sr_2_RuO_4_. **d** Ru-*L*_2,3_ XAS spectra of Sr_2_RuO_4_ and ADT-Sr_2_RuO_4_. Insert is the magnified Ru-*L*_3_ spectra of Sr_2_RuO_4_ and ADT-Sr_2_RuO_4_. **e** HAADF-STEM image and the corresponding EDX elemental mapping of ADT-Sr_2_RuO_4_. **f** EDX spectra at the position in points 1 and 2 in (**e**)

It is interesting to note that the HER activity is further improved after long-term stability test (Supplementary Fig. 15). During the chronopotentiometry test at −10 mA cm^−2^, the overpotential gradually decreases in the first ~5 h and then becomes stable during the next 25 h, indicating an activation process with self-improved activity for Sr_2_RuO_4_^47–50^. The activation process was further verified by accelerated durability test (ADT) with continuous cycling in the HER potential window (Fig. 4a), showing that the overpotential decreases distinctly. To obtain a deeper understanding and to seek the origin of the activation phenomenon during the electrocatalytic process, the Sr_2_RuO_4_ catalyst after ADT of 1000 cycles (denoted as ADT-Sr_2_RuO_4_) was carefully characterized. Firstly, we checked the change in ECSA of Sr_2_RuO_4_ and ADT-Sr_2_RuO_4,_ which is estimated from the double-layer capacitances by CV method (Supplementary Fig. 8e and Fig. 25). Figure 4b displays the current density differences as a function of scan rate of Sr_2_RuO_4_ and ADT-Sr_2_RuO_4_, and the result displays that the ECSA of ADT-Sr_2_RuO_4_ is nearly two times larger than that of pristine Sr_2_RuO_4_. The evident increase of ECSA after ADT may be associated with the change in the microstructure and composition of the catalysts^47–50^. The XRD patterns (Fig. 4c) shows that there is no apparent variation in the peak pattern and position between Sr_2_RuO_4_ and ADT-Sr_2_RuO_4_, revealing the unchanged bulk crystal structure of Sr_2_RuO_4_ during ADT. XAS technique was adopted to investigate the surface electronic structure of the transition metal. As displayed in Fig. 4d, a 0.2-eV shift of Ru-*L*_2,3_ edge toward low photo energy was observed for ADT-Sr_2_RuO_4_ relative to Sr_2_RuO_4_, which implies partial reduction of surface Ru^4+^. Moreover, the surface morphology was also examined and Supplementary Fig. 26 shows TEM images of the surface regions of ADT-Sr_2_RuO_4_. It is clearly seen that some nanoparticles with a radius of 10–15 nm are dispersed on the ADT-Sr_2_RuO_4_ surface. The lattice fringe with lattice space of 0.23 nm corresponds to the (001) plane of hexagonal Ru, suggesting that these nanoparticles are composed of metallic Ru in accordance with the XAS result. The high-angle annular dark-field scanning transmission electron microscopy (HAADF-STEM) image (Fig. 4e) together with the energy-dispersive X-ray spectroscopy (EDX) elemental mapping and point spectra (Fig. 4f) further collectively confirm the formation of metallic Ru nanoparticles and oxide on the surface of ADT-Sr_2_RuO_4_. In conjunction with the above XRD, XAS, TEM, STEM, EDX and electrochemical results, we can conclude that the further-improved activity for Sr_2_RuO_4_ through electrochemical activation mainly originates from the interface synergy between in situ formation of Ru nanoparticle and oxide substrate over the surface^51–53^ as well as increased ECSA. The complete picture of interface synergy between these two components requires a future in-depth work.

## Discussion

In summary, we have demonstrated that that layered RP Sr_2_RuO_4_ oxide has very high electrocatalytic activity for HER in alkaline media. Remarkably, when evaluated in 1 M KOH solution, the catalyst exhibits an onset overpotential of only 3 mV, low overpotential of 61 mV at 10 mA cm^−2^, low Tafel slope of 51 mV dec^−1^, large exchange current density of 0.898 mA cm^−2^, and high TOF values (e.g., 0.90 s^−1^ at overpotential of 100 mV), which is comparable to a commercial Pt/C catalyst and competitive with the best Pt-free electrocatalysts ever reported. Such excellent HER activity of Sr_2_RuO_4_ is attributed mainly to a synergistic effect associated with the layered RP structure, in which the SrO-terminated surface cleaved the in rock-salt layer promotes water dissociation, and the apical oxygen site in the perovskite layer facilitates favorable hydrogen adsorption, as confirmed by experiments and theoretical calculations. The favorable hydrogen adsorption for Sr_2_RuO_4_ on oxygen site is closely associated with the d-band center of Ru and Ru−O bond length. Moreover, the HER activity of Sr_2_RuO_4_ can be further enhanced by interface synergy between in situ formation of Ru nanoparticle and oxide substrate over the surface as well as increased active sites during the long-term stability test, which are substantiated by XRD, XAS, TEM, STEM, EDX and electrochemical analysis. This work not only demonstrates a new family of layered RP Sr_2_RuO_4_ oxide as an efficient HER catalysts, but also opens a new avenue for the development of next-generation high-performance electrocatalysts through unusual synergistic effects in the crystal structure.

## Methods

### Catalyst synthesis

The Sr_2_RuO_4_ was synthesized by traditional solid-state reaction route. Taking the synthesis of Sr_2_RuO_4_ as an example, stoichiometric amounts of SrCO_3_ and RuO_2_ were weighed and mixed using a high-energy ball mill (Planetary Mono Mill, Pulverisette 6, Fritsch) in ethanol media under 400 rpm for 1 h. The homogeneously dispersed mixture was then dried and calcined at 1200 °C in air for 20 h to form the Sr_2_RuO_4_ powders. SrRuO_3_ was also prepared by the identical procedure as Sr_2_RuO_4_ with the exception of the distinction of raw materials and their contents. The RuO_2_ powder used in this study was purchased from Alfer Aesar and the commercial Pt/C catalyst was purchased from Johnson Matthey Company.

### Basic characterizations

The crystal structure of the as-prepared powders was studied by room temperature XRD (Rigaku Smartlab) using filtered Cu-Kα radiation (*λ* = 1.5418 Å) at a tube voltage of 40 kV and current of 40 mA. XRD patterns were collected by step scanning with an interval of 0.02° in the 2*θ* range of 10–90°. More detailed structural information (e.g., the space group and lattice parameters) was attained by Rietveld refinements using the GSAS-EXPGUI package. HRTEM images and the corresponding SAED patterns were obtained on a transmission election microscope (TEM, FEI Tecnai G2T20) at an accelerating voltage of 300 kV. HAADF-STEM images along with the corresponding EDX compositional line profiles were attained on a field-emission transmission electron microscope (FEI Tecnai G2 F30 STWIN) equipped with an EDX analyzer at 200 kV. SEM images were obtained using a Hitachi S-4800 scanning electron micro-analyzer. Nitrogen adsorption isotherms were tested under 77 K (BELSORP II) and the specific surface areas were calculated by the BET methods. An Agilent (7820 A) gas chromatography system equipped with an HP-plot molecular sieve (5 Å) column and a thermal conductivity detector was employed to obtain gas chromatography data. Hydrogen was detected using a long (24 m length) column with a nitrogen carrier gas. The surface element states were probed using an XPS (PHI5000 VersaProbe equipped with an Al Kα X-ray source) and public XPSPEAK software package was used to fit the obtained data. The synchrotron-based measurements were carried out using the soft X-ray spectroscopy beamline at the Taiwan Synchrotron. The Ru-*L*_3_ XAS spectra were measured in the total-electron-yield mode. Ru-*L*_3_ XAS spectra of Ru-based standard references (RuCl_3_, Sr_2_RuO_4_, and Sr_2_GdRuO_6_) were collected under the same conditions.

### Electrode preparation

Working electrodes for HER measurements were prepared by a controlled drop-casting method including an RDE comprising a glassy carbon (GC) electrode with 0.196 cm^2^ (Pine Research Instrumentation), which was prepolished with aqueous alumina suspension. To eliminate the electrode conductivity restriction within thin film working electrodes, the catalysts in this study were mixed with as-obtained conductive carbon (Super P Li) at a 1:1 mass ratio. Briefly, a 5 μL aliquot of the catalyst ink, which was prepared by sonication of a mixture of 10 mg of oxide powder and 10 mg of conductive carbon dispersed in 1 mL ethanol and 100 μL of 5 wt% Nafion solution for at least 1 h, was dropped on the GC surface, generating an approximate catalyst loading of 0.464 mg_total_ cm^−2^ (0.232 mg_oxide_ cm^−2^) and was left to dry before the electrochemical tests. Working electrode containing commercial 20 wt% Pt/C catalyst was prepared according to the following procedures. Ten milligrams Pt/C and 100 μL of 5 wt% Nafion solution were ultrasonically dispersed in 1 mL of ethanol to form a homogeneous ink. Five microliters of the homogeneous ink was transferred onto the surface of the GC substrate, generating an approximate catalyst loading of 0.232 mg_total_ cm^−2^ (0.0464 mg_Pt_ cm^−2^).

### Electrochemical measurements

HER performance of catalysts in alkaline or acidic solutions was assessed on a CHI 760D electrochemistry workstation at room temperature in a standard three-electrode electrochemical cell (Pine Research Instrumentation) in an RDE configuration. Catalysts cast on RDE, graphite rod and Ag|AgCl (3.5 M KCl) were used as the working electrode, counter electrode, and reference electrode, respectively. Throughout the measurement, the RDE electrode was rotated at 1600 rpm to remove the bubbles formed at the electrode surface. The electrolyte solution was bubbled with Ar for ~30 min before HER tests and maintained under Ar atmosphere during the test period. HER polarization curves were recorded from −0.8 to −1.6 V vs. Ag|AgCl in 1 M KOH solutions and from 0 to −0.6 V vs. Ag|AgCl in 0.5 H_2_SO_4_ solutions. *IR* compensation for all polarization curves in this study was performed unless noted otherwise. Tafel plots were obtained by replotting the polarization curves as overpotential (*η*) versus the logarithm of current density (log |*J*|). Extrapolating the Tafel plots to the overpotential of 0 V to attain the exchange current density (*j*_0_). Accelerated durability tests of catalysts were conducted through continuous potential cycling ranged from −0.8 to −1.4 V vs. Ag|AgCl for 1000 cycles at a scan rate of 100 mV s^−1^. The chronopotentiometry tests were performed at a constant cathodic current density of 10 mA cm^−2^. CV tests were conducted in the potential range between −0.8 and −0.9 V vs. Ag|AgCl (where no faradic current was observed) with different sweeping rates of 20, 40, 60, 80, and 100 mV s^−1^ to measure the electrochemical double-layer capacitance (*C*_dl_). The slopes obtained from the plots as the halves of the positive and negative current density differences at the center of the scanning potential range (i.e., −0.85 V) versus scan rates are the *C*_dl_ values.

### Computational methods

Electronic structure calculations were performed using the Vienna ab initio Simulation Package (VASP)^54,55^. Exchange and correlation were described using the generalized gradient approximation with the Perdew−Burke−Ernzerhof functional^56^. The projector augmented wavefunction method^57^ was used to describe pseudopotentials. Van der Waals correction was employed in all calculations^58^. A kinetic energy cut-off of 500 eV was used. Unit cells of Ru, Pt, RuO_2_, SrRuO_3_, and Sr_2_RuO_4_ were fully relaxed, and the resulting structures were cleaved to construct symmetric slabs which were sandwiched in about 20 Å of vacuum. Hydrogen adsorption was investigated by placing H on various sites on the metal and oxides surfaces, and the binding energy was calculated as:

1 $${\mathrm{\Delta }}E_{\mathrm H} = E_{{\mathrm H}^ \ast } - E_{{\mathrm {bare}}} - 1/2E_{{\mathrm H}_2},$$

where $E_{{\mathrm H} \ast }$, $E_{{\mathrm {bare}}}$, and $E_{{\mathrm H}_2}$ are the total energies of an adsorbed hydrogen, the bare surface and the energy of a hydrogen molecule, respectively. Free energies ${\mathrm{\Delta }}G_{{\mathrm H}^ \ast }$, were obtained by adding the zero-point energy (ZPE) and entropic correction to the binding energy such as ${\mathrm{\Delta }}G_{{\mathrm H}^ \ast } = {\mathrm{\Delta }}E_{\mathrm H} + {\mathrm{\Delta }}E_{{\mathrm {ZPE}}} - T{\mathrm{\Delta }}S$. The total correction ${\mathrm{\Delta }}E_{\mathrm{ZPE}} - T{\mathrm{\Delta }}S$ was estimated to be 0.24 eV for metals and oxides and this value is used consistently throughout^59^. Metal d-band centers were determined by evaluating the centroid of the projected DOS including valence and conduction states relative to the Fermi level. Water dissociation barriers were calculated using the climbing image nudged elastic band^60^ (CI-NEB) using five images and a force tolerance of 0.02 eV/Å. The Volmer−Heyrovsky pathway of HER in alkaline media (2H_2_O + 2e^−^ → H_2_ + 2OH^−^) proceeds through two electrochemical elementary steps and two electrons involved (H_2_O + * + e^−^ → H* + OH^−^; H* + H_2_O + e^−^ → H_2_ + * + OH^−^; where the * denotes the catalyst surface). The effect of pH can be accounted for within the computational hydrogen electrode formalism by including the changes due to *k*_B_*T*ln10*pH (*k*_B_ is Boltzmann’s constant) for which the chemical potential for H^+^ + e^−^ = 1/2H_2_ − *k*_B_*T*ln10*pH. A pH = 14 corresponding to fully alkaline conditions results in an equilibrium potential of 0.83 V. The energy of *G*(OH^−^) can be obtained from *G*(H_2_O) and *G*(H^+^) via *G*(OH^−^) = *G*(H_2_O) − *G*(H^+^) = *G*(H_2_O) − *G*(1/2H_2_) + *k*_B_*T*ln10*pH^41^.

## Supplementary information

Supplementary Information

## Data Availability

The data that support the findings of this study are available from the corresponding authors upon request.
