# Supplementary material for: Unusual synergistic effect in layered Ruddlesden−Popper oxide enables ultrafast hydrogen evolution
Source: Nat Commun. 2019 Jan 11;10:149. doi: 10.1038/s41467-018-08117-6 (PMC6329747; doi:10.1038/s41467-018-08117-6)
Supplement: Supplementary file 1 — Supplementary Information [file 41467_2018_8117_MOESM1_ESM.pdf]

# Unusual synergistic effect in layered Ruddlesden-Popper oxide enables ultrafast hydrogen evolution

**Zhu *et al.***

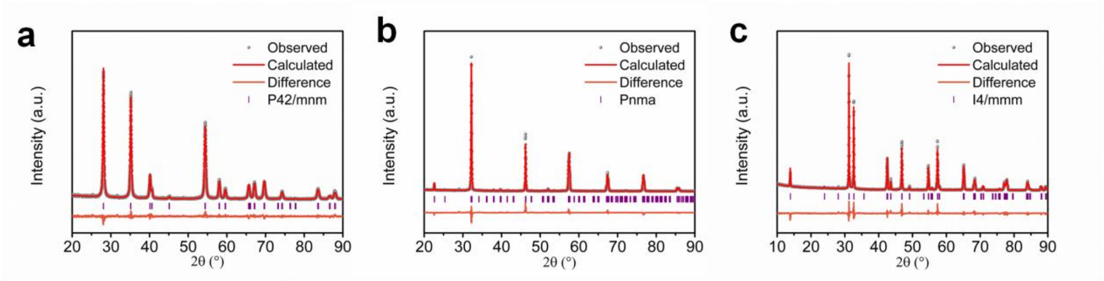


**Supplementary Figure 1. Rietveld refinement of XRD patterns.** **a** $RuO_2$, **b** $SrRuO_3$ and **c** $Sr_2RuO_4$. Observed (gray circles), calculated (red solid line), differences (orange line, bottom) and calculated Bragg positions (vertical purple bars) are presented.

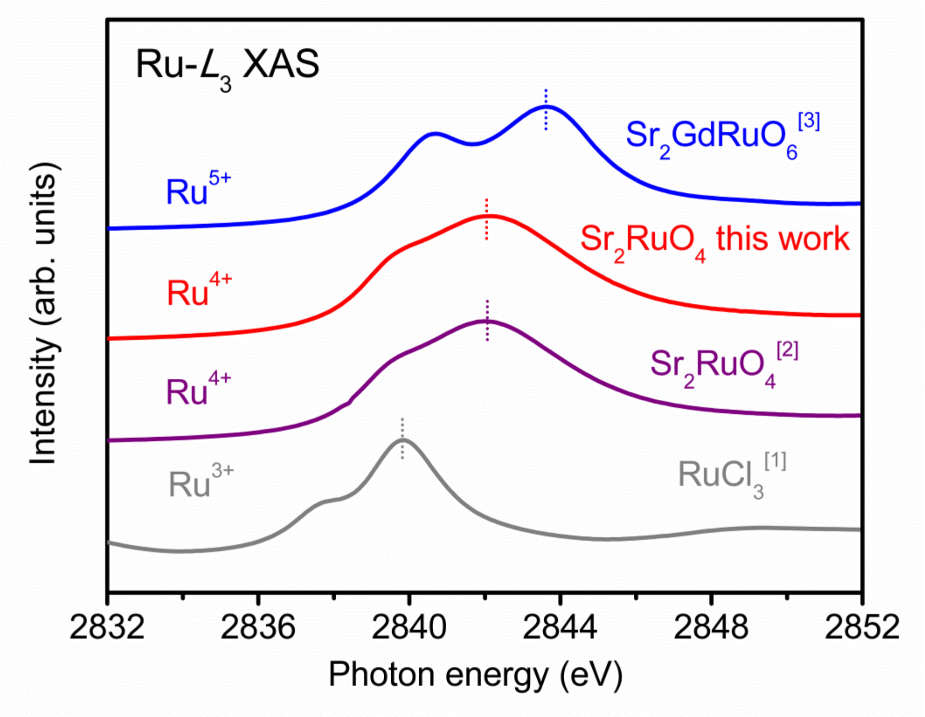


**Supplementary Figure 2.** Ru-$L_{2,3}$ XAS spectra of $Sr_2RuO_4$ in comparison with the standard $RuCl_3$ ($Ru^{3+}$)[1], $Sr_2RuO_4$ ($Ru^{4+}$)[2] and $Sr_2GdRuO_6$ ($Ru^{5+}$)[3] reference materials.

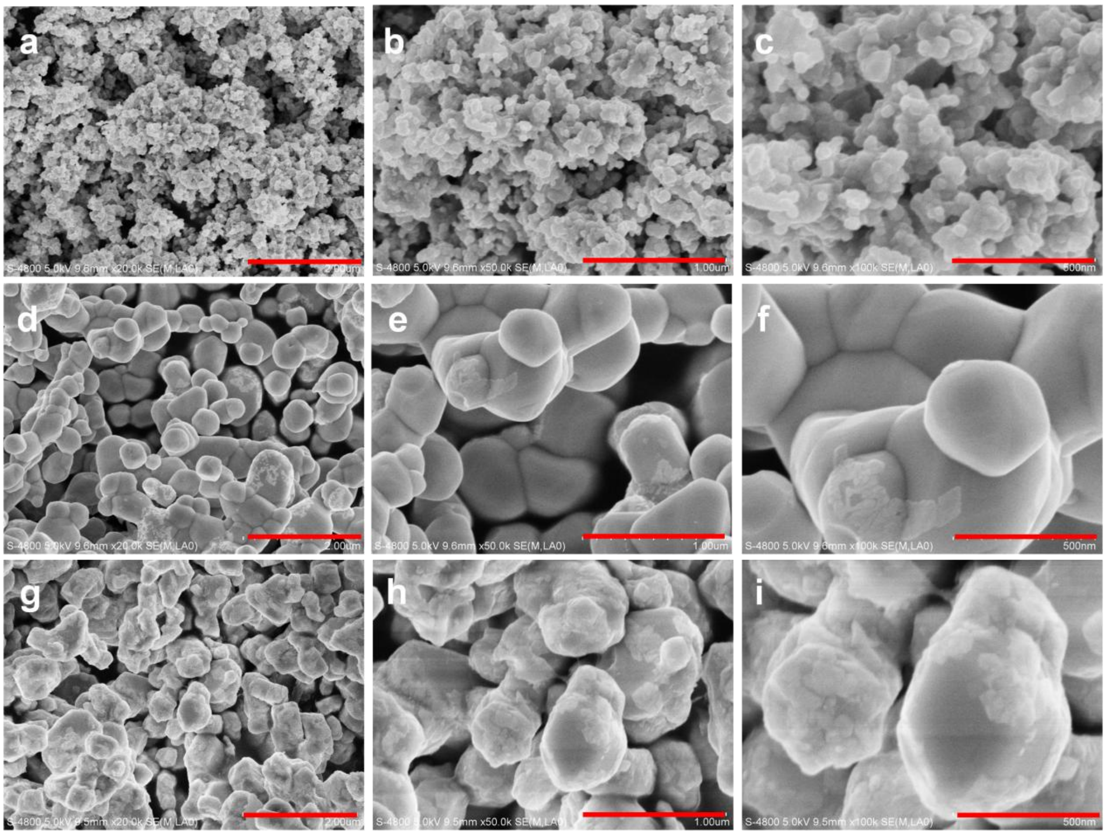


**Supplementary Figure 3. SEM images with different magnifications**. **a-c** $RuO_2$, **d-f** $SrRuO_3$ and **g-i** $Sr_2RuO_4$. Scale bar in **a, d, j** is 2 μm, in **b, e, h** is 1 μm,and in **c, f, i** is 500 nm.

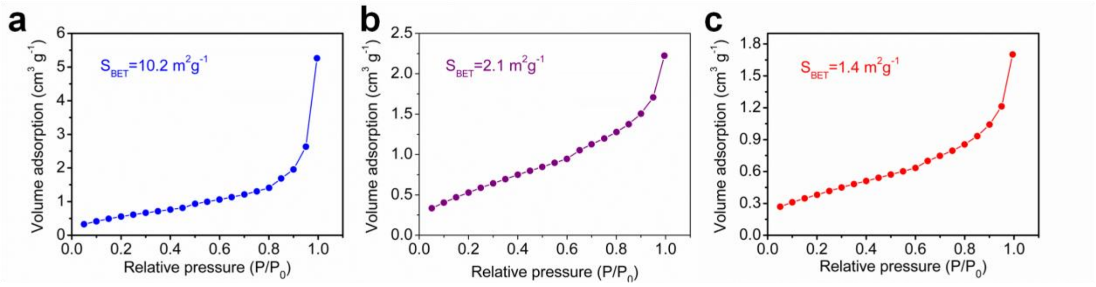


**Supplementary Figure 4. $N_2$ adsorption isotherm. a** $RuO_2$, **b** $SrRuO_3$ and **c** $Sr_2RuO_4$.

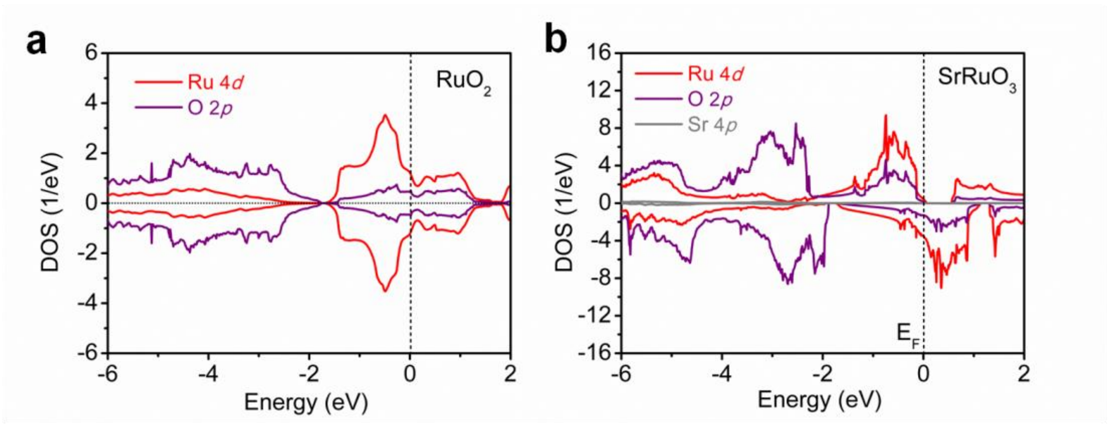


**Supplementary Figure 5. DOS calculation**. **a** $RuO_2$ and **b** $SrRuO_3$.

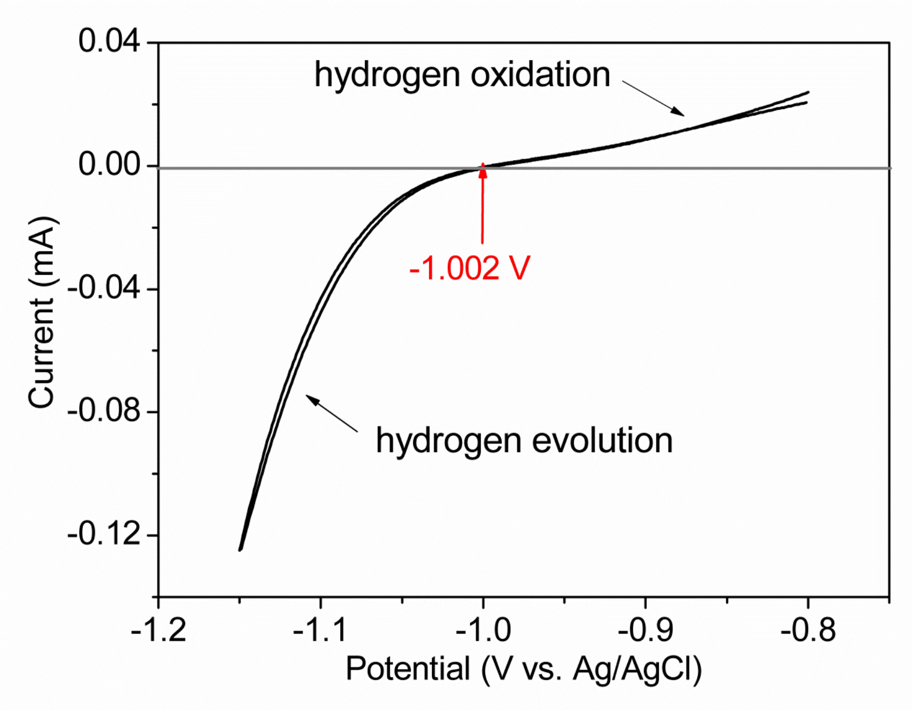


**Supplementary Figure 6.** Potential calibration of the Ag/AgCl reference electrode in 1 M KOH solution. The calibration was performed in a high purity hydrogen-saturated electrolyte with a platinum rotating disk electrode (PINE, 4 mm diameter, 0.126 $cm^2$) as the working electrode. Cyclic voltammetry (CV) was run at a scan rate of 1 mV $s^{-1}$, and the average of the two potentials at which the current crossed zero was taken to be the thermodynamic potential for the hydrogen electrode reaction. In 1 M KOH, $E_{RHE} = E_{Ag/AgCl}$ +1.002 V.

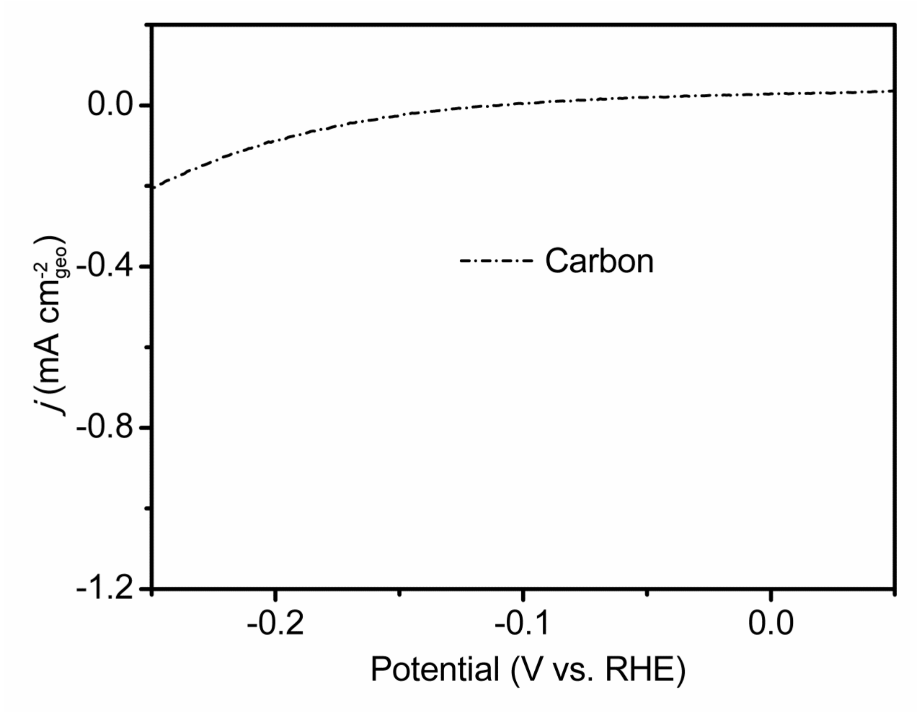


**Supplementary Figure 7.** Polarization curve of conductive carbon in an Ar-saturated 1 M KOH solution.

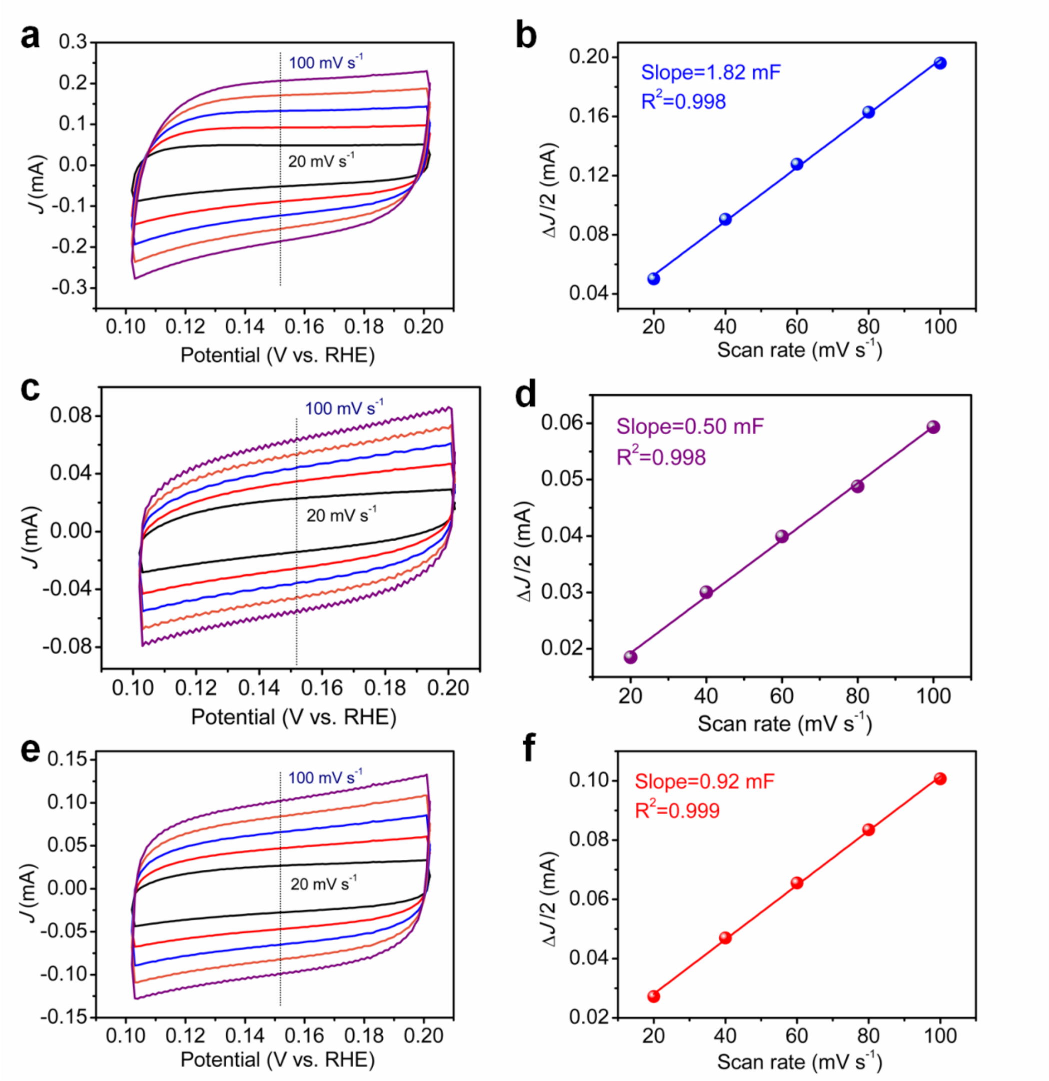


**Supplementary Figure 8. ECSA estimination determined from $C_{dl}$.** CV measurements in a non-faradic current region (0.102-0.202 V vs. RHE, no *iR*-corrected) at scan rates of 20, 40, 60, 80 and 100 mV $s^{-1}$ of **a** $RuO_2$, **c** $SrRuO_3$ and **e** $Sr_2RuO_4$ catalysts in 1 M KOH. Linear fitting of the capacitive currents versus CV scan rates for **b** $RuO_2$, **d** $SrRuO_3$ and **f** $Sr_2RuO_4$ catalysts.

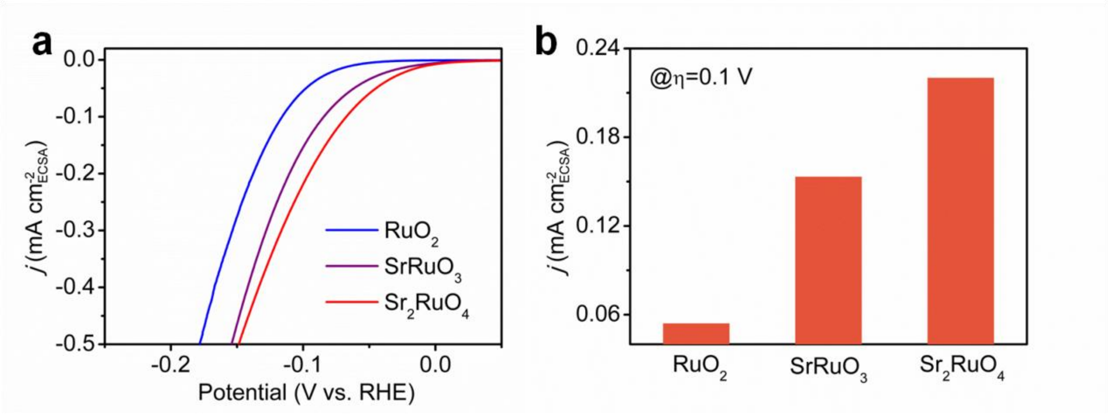


**Supplementary Figure 9. Specific activity normalized to ECSA**. **a** Specific activity as a function of potential. **b** Specific activity at the overpotential of η=0.1 V.

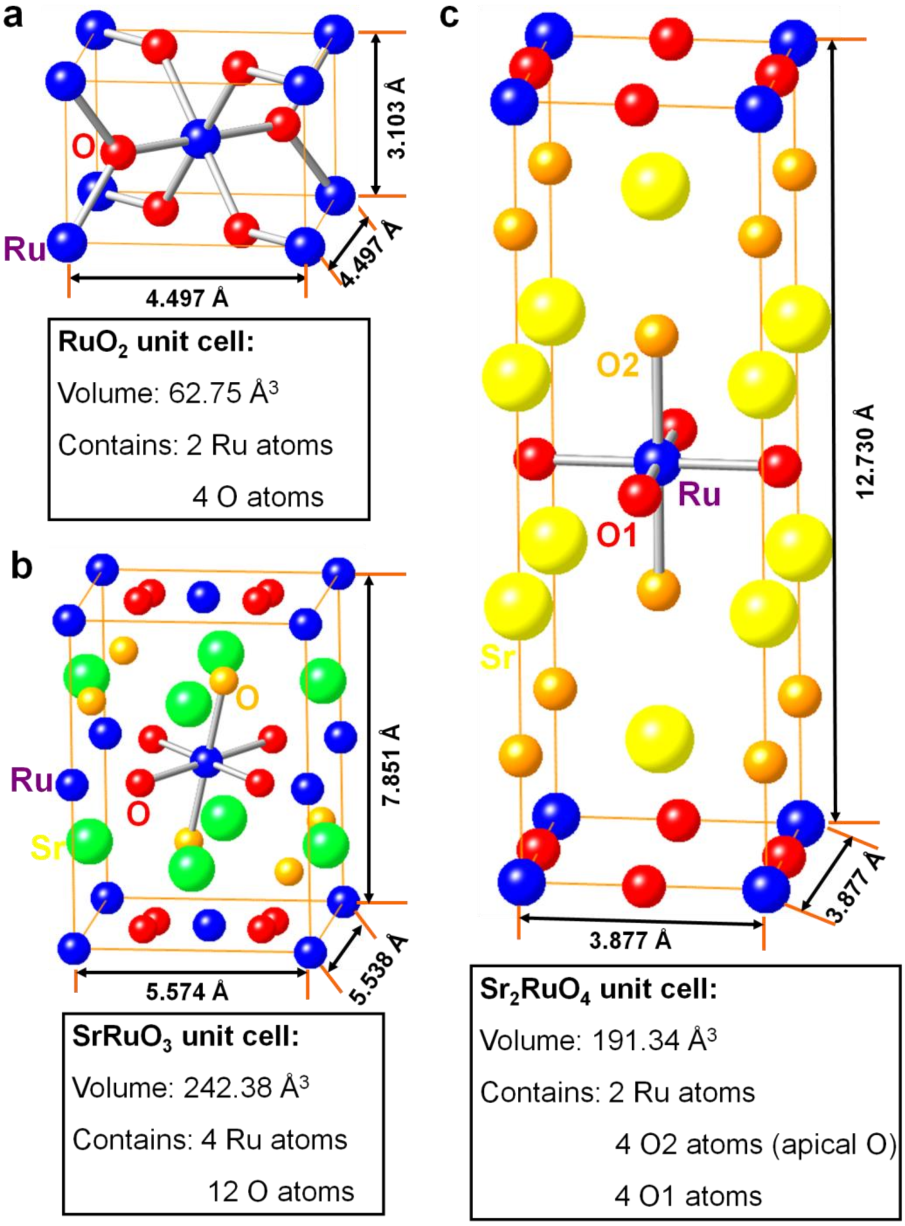


**Supplementary Figure 10. Unit cell of catalysts**. **a** $RuO_2$, **b** $SrRuO_3$ and **c** $Sr_2RuO_4$ unit cell.

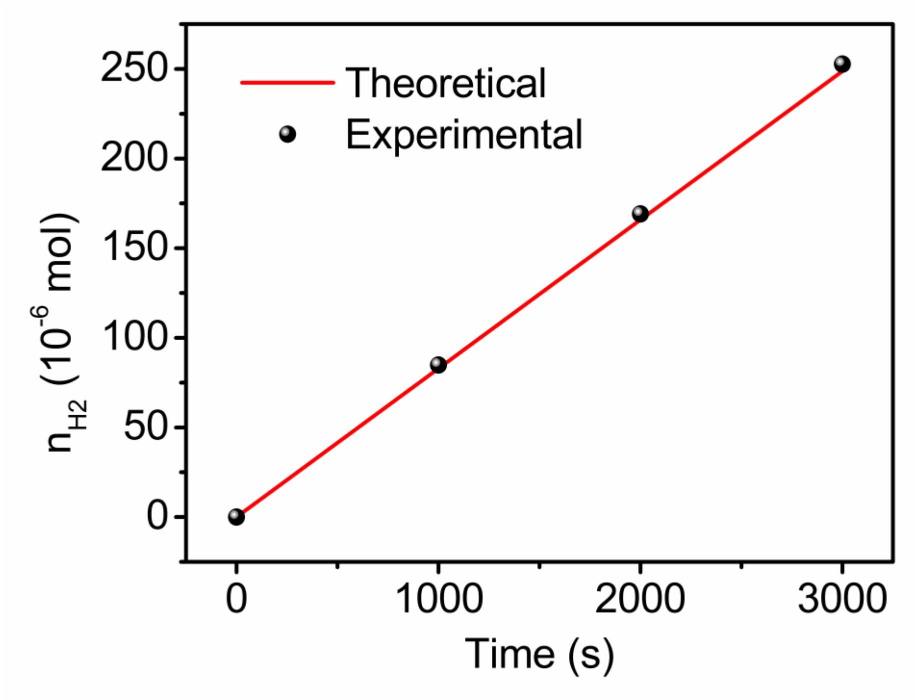


**Supplementary Figure 11.** The amount of theoretically calculated (red line) and experimentally measured (black balls) hydrogen versus time for $Sr_2RuO_4$ at -1.2V vs. Ag/AgCl for 3000 s.

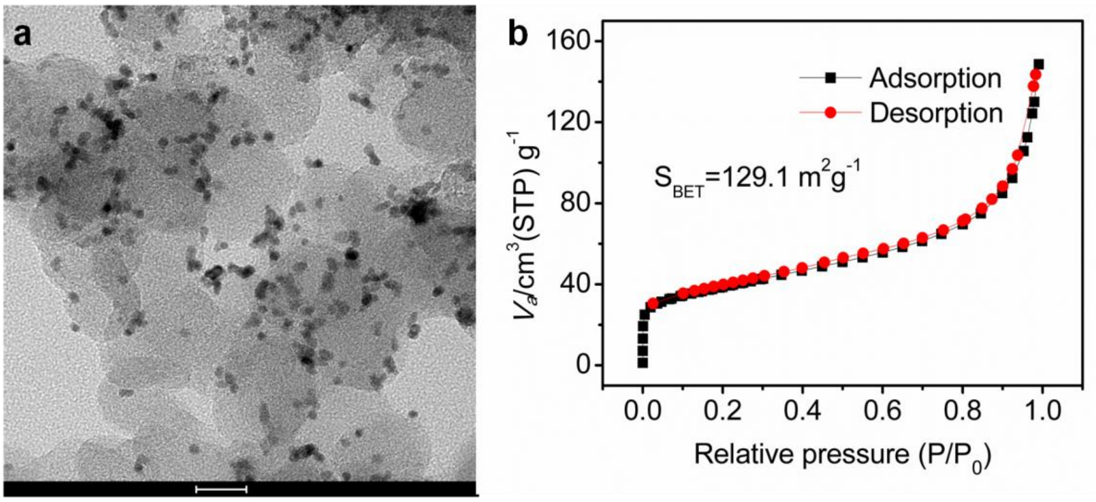


**Supplementary Figure 12. Commercial 20 wt% Pt/C catalysts (Johnson Matthey Company)**. **a** TEM image. The small Pt particles are ~5 nm. **b** $N_2$ adsorption-desorption isotherm. Scale bar in **a** is 20 nm.

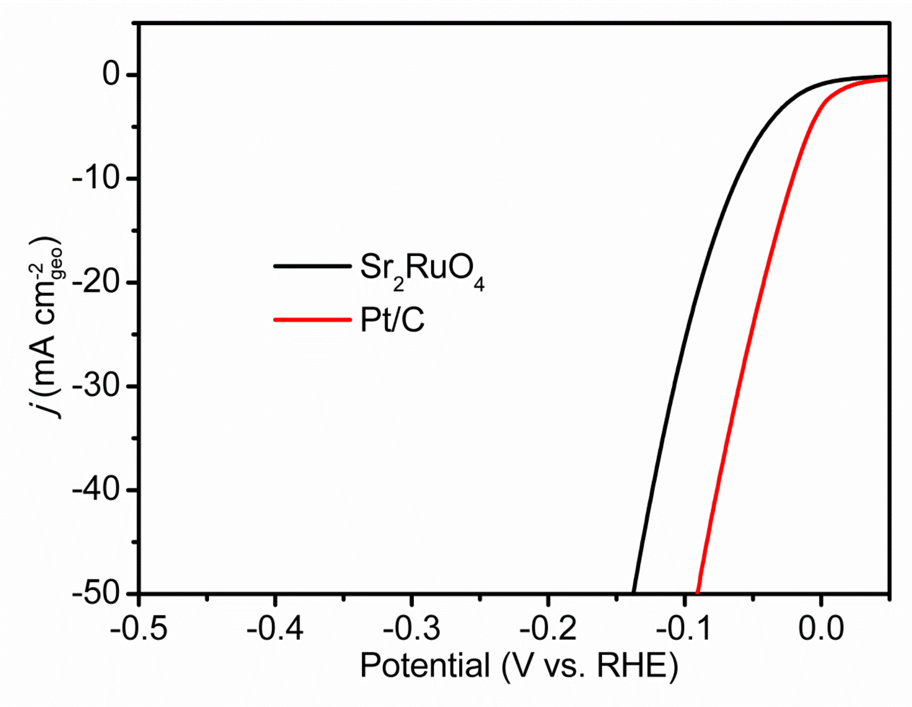


**Supplementary Figure 13.** Polarization curve of $Sr_2RuO_4$ and commercial Pt/C catalysts in an Ar-saturated 1 M KOH solution.

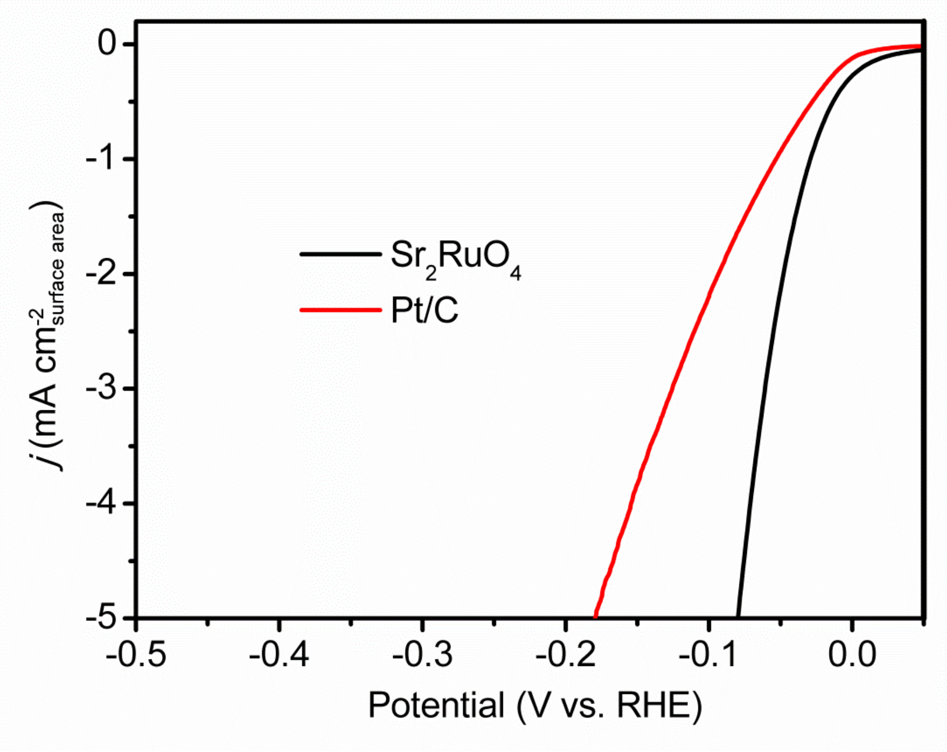


**Supplementary Figure 14.** Specific activity normalized to surface area of $Sr_2RuO_4$ and commercial Pt/C catalysts as a function of potential. The surface area of the Pt nanoparticles supported on carbon could be estimated using a spherical geometry approximation[4,5]: $A_s$=6/($\rho d$), where $\rho$ is the bulk density ($\rho_{Pt}$=21.45 g $cm^{-3}$), $d$ represents the average size of the nanoparticles particles (for Pt in Pt/C, $d$ equals to ~5 nm according to the TEM image); thus values of $A_s$ were calculated to be 55.9 $m^2$ $g^{-1}$ for Pt in Pt/C. The surface area of $Sr_2RuO_4$ oxide was estimated to be 1.4 $m^2$ $g^{-1}$ via BET measurement.

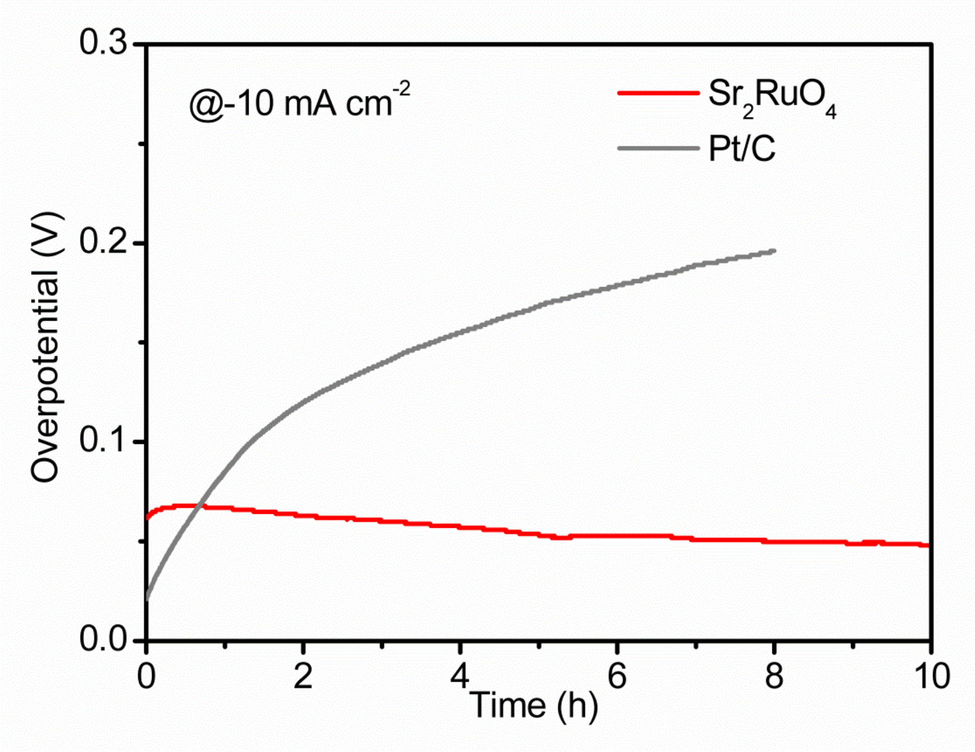


**Supplementary Figure 15.** Chronopotentiometry curves of $Sr_2RuO_4$ and Pt/C catalysts at a constant cathodic current density of -10 mA cm$^{-2}$.

| | | |
|---|---|---|
| 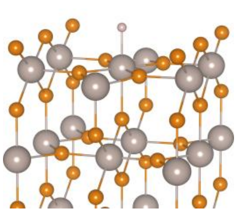 | 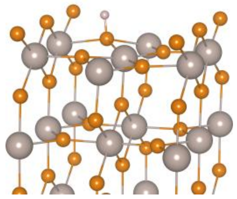 | 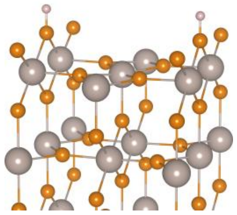 |
| H@Ru$^{cus}$<br>$\Delta G_{H^*} = 0.48\ eV$ | H@O<br>$\Delta G_{H^*} = 0.36\ eV$ | H@O$^{bridge}$<br>$\Delta G_{H^*} = -0.8\ eV$ |

**Supplementary Figure 16. Structural models and calculated sites of $RuO_2$ (110) for H* adsorption.** Gray balls represent Ru atoms, orange balls represent O atoms, light gray represent H atom. the $RuO_2$ (110) surface exposes coordinatively unsaturated Ru atom, terminal O atom and bridge O atom, denoted as Ru$^{cus}$, O and O$^{bridge}$, respectively.

| | |
|---|---|
| 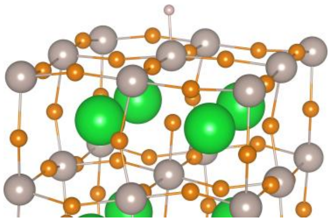 | 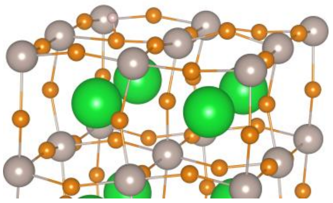 |
| H@Ru<br>$\Delta G_{H^*} = -0.23\ eV$ | H@O<br>$\Delta G_{H^*} = -0.47\ eV$ |

**Supplementary Figure 17. Structural models and calculated sites of $SrRuO_3$ (010) for H* adsorption.** Gray balls represent Ru atoms, orange balls represent O atoms, green balls represent Sr atoms, light gray represent H atom.

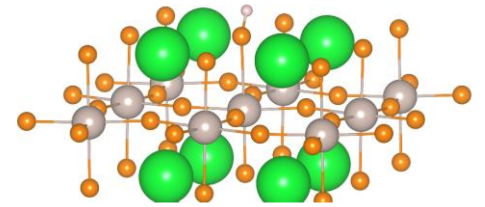

| H@O<br>$\Delta G_{H^*} = -0.08\ eV$ |
|---|

**Supplementary Figure 18. Structural models and calculated sites of $Sr_2RuO_4$ (001) for H* adsorption.** Gray balls represent Ru atoms, orange balls represent O atoms, green balls represent Sr atoms. light gray represent H atom.

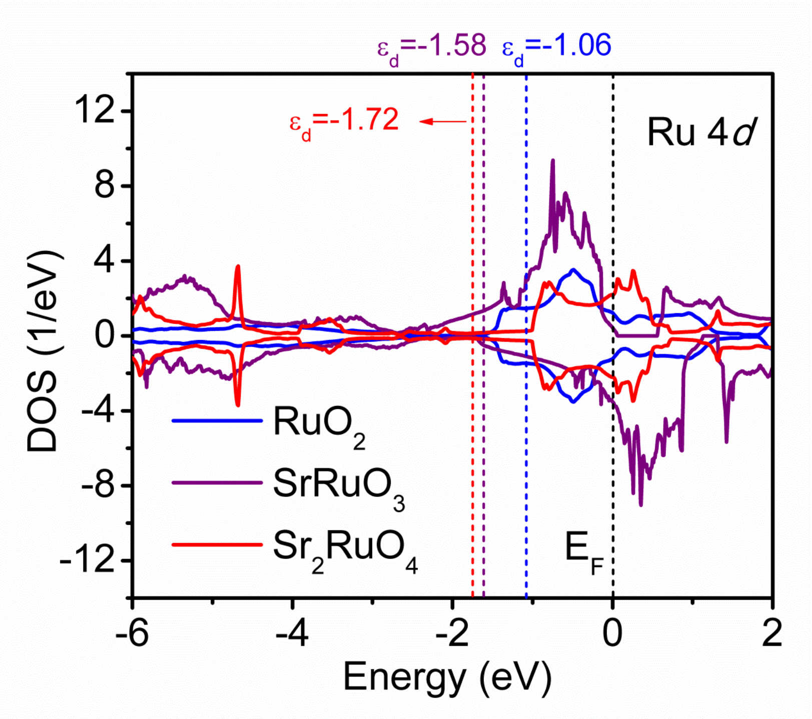


**Supplementary Figure 19.** Projected density of states (PDOS) of Ru 4*d* on $RuO_2$, $SrRuO_3$ and $Sr_2RuO_4$.

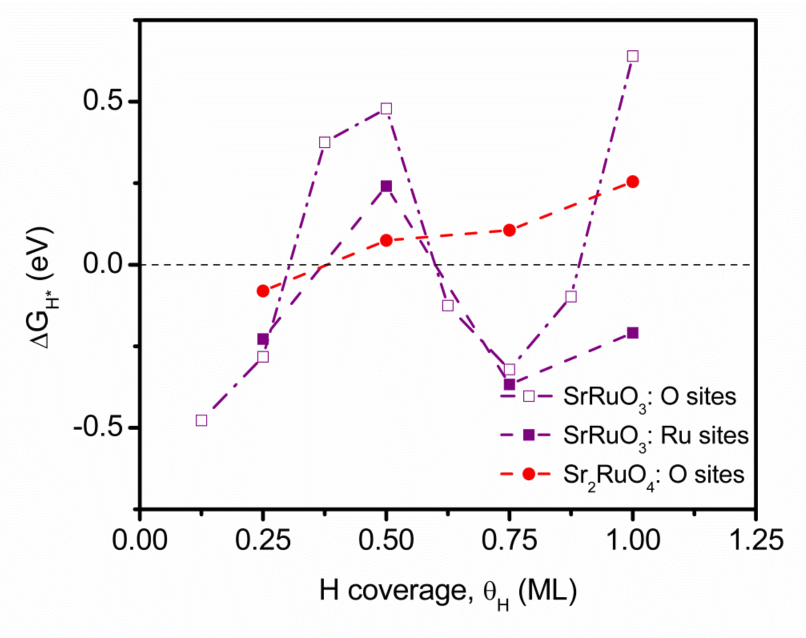


**Supplementary Figure 20. Coverage effect of H on $\Delta G_{H*}$ for the SrO termination of $Sr_2RuO_4$ and the $RuO_2$ termination of $SrRuO_3$.** As can be seen, $\Delta G_{H^*}$ is very sensitive to coverage in $SrRuO_3$ displaying a somewhat complex trend. On the other hand, $\Delta G_{H^*}$ in $Sr_2RuO_4$ displays a nearly monotonic increase with coverage. The result indicates that $Sr_2RuO_4$ can achieve higher converges with lower differential adsorption energies. This is not the case with $SrRuO_3$ where there is a sharp rise in the differential adsorption energy for converges > 0.25 ML. However, it is noticeable that $\Delta G_{H^*}$drops again at $\theta_H$ = 0.5 ML. This behaviour stems in part from the complex structural and electronic rearrangements at the $RuO_2$ terminated $SrRuO_3$.

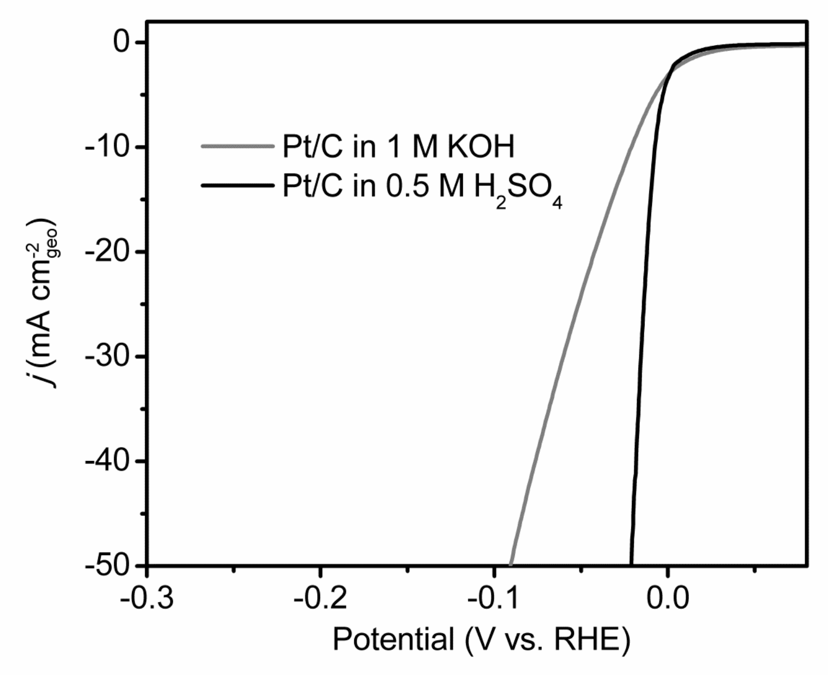


**Supplementary Figure 21.** Polarization curve of Pt/C catalyst in Ar-saturated 1 M KOH and 0.5 M $H_2SO_4$ solutions.

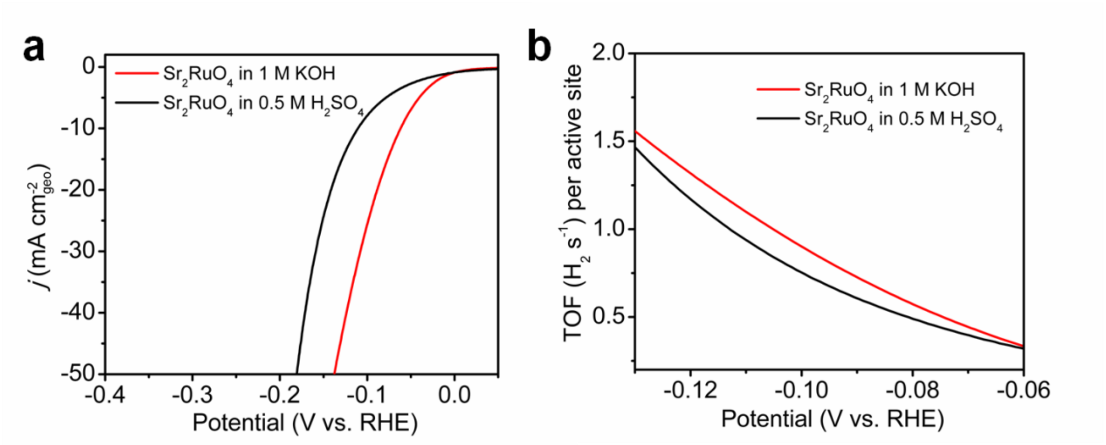


**Supplementary Figure 22. HER activity of $Sr_2RuO_4$ in alkaline and acid media. a** Polarization curve of $Sr_2RuO_4$ in Ar-saturated 1 M KOH and 0.5 M $H_2SO_4$ solutions. **b** The relationship between TOF and the measured potentials for $Sr_2RuO_4$ in Ar-saturated 1 M KOH and 0.5 M $H_2SO_4$ solutions.

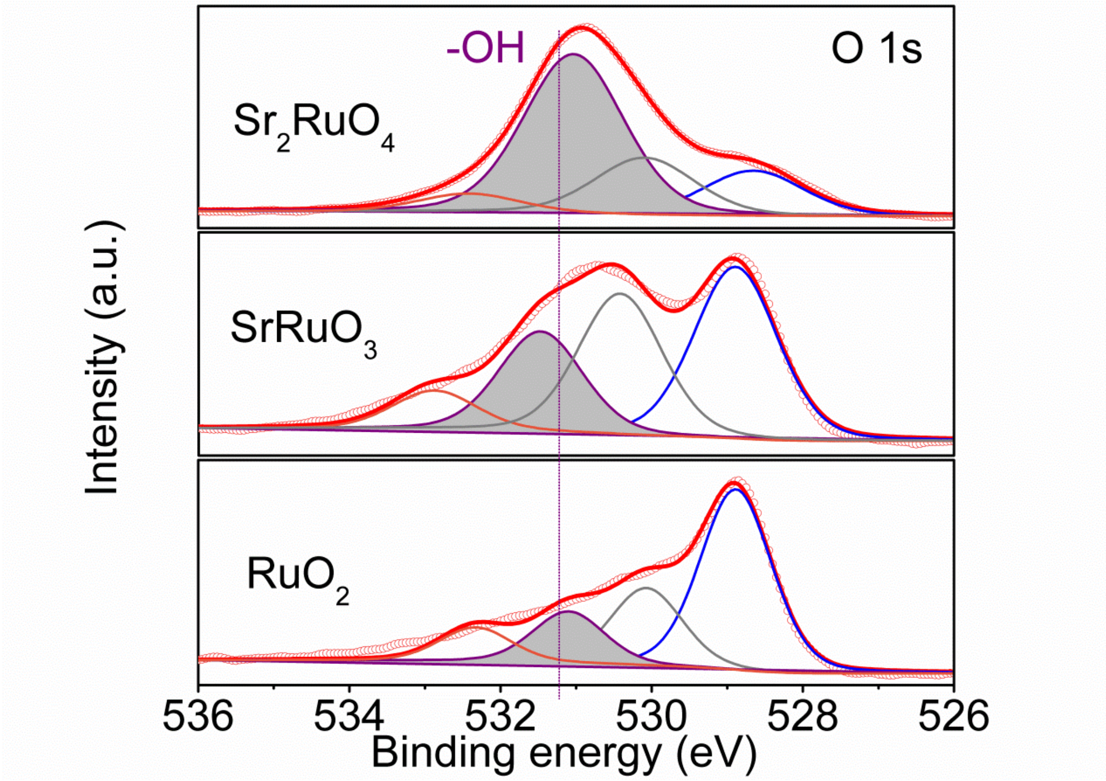


**Supplementary Figure 23.** XPS spectra of O 1s species on the surface of $Sr_2RuO_4$, $SrRuO_3$ and $RuO_2$.

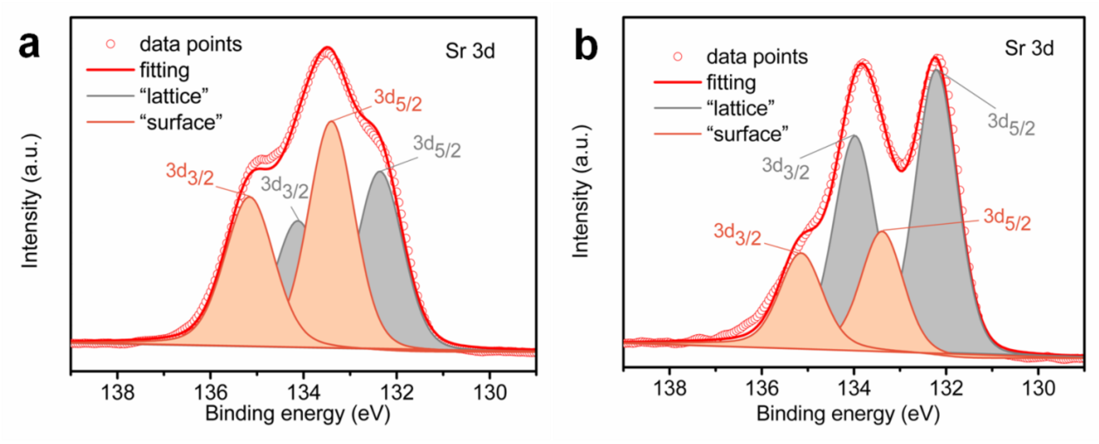


**Supplementary Figure 24. XPS Sr 3d spectra**. **a** $Sr_2RuO_4$ and **b** $SrRuO_3$. Each spectrum can be well fitted considering the presence of two different chemical environments for the Sr cations. The low-energy component is referred to as lattice Sr ($Sr_L$), and the high-energy component is referred to as surface Sr ($Sr_S$). $Sr_S$ indicates the formation of hydroxide surface species[6,7]. The surface Sr species of $Sr_2RuO_4$ is mainly $Sr_S$, while the surface Sr species of $Sr_2RuO_4$ is mainly $Sr_L$.

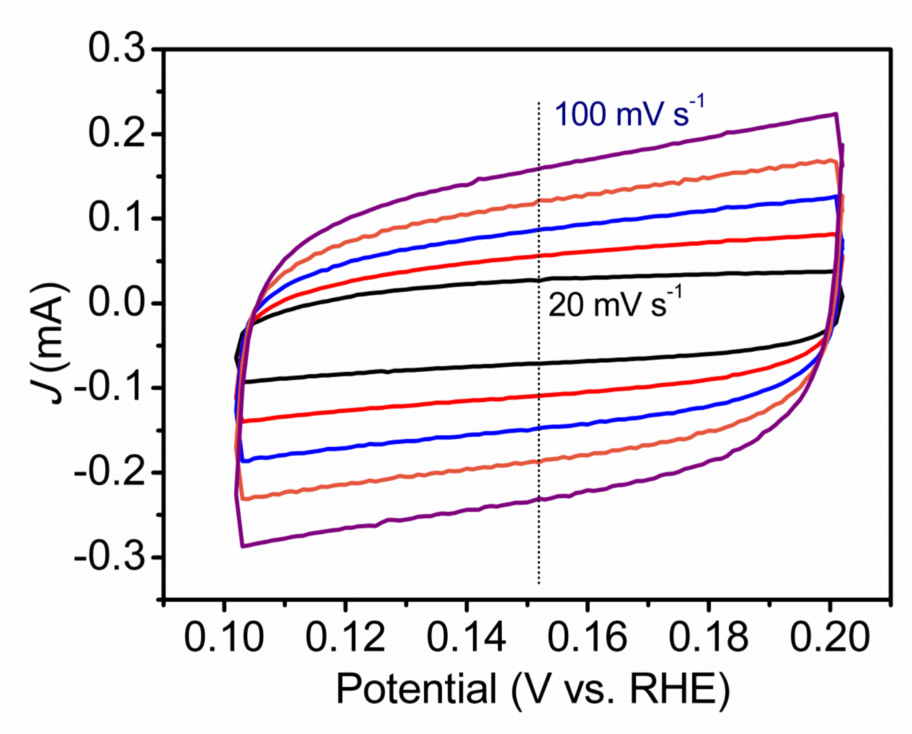


**Supplementary Figure 25.** CV measurements in a non-faradic current region (0.102-0.202 V vs. RHE, no *iR*-corrected) at scan rates of 20, 40, 60, 80 and 100 mV $s^{-1}$ of ADT-$Sr_2RuO_4$ in 1 M KOH.

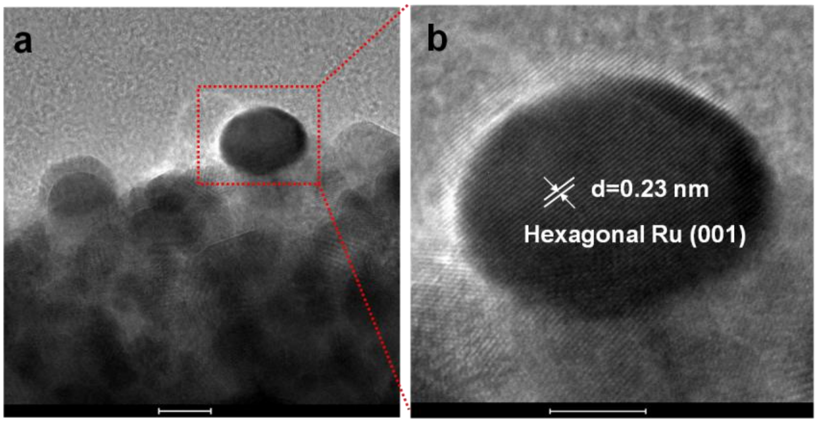


**Supplementary Figure 26.** TEM images with different magnifications of ADT-$Sr_2RuO_4$. Scale bar in **a** is 10 nm and in **b** is 5 nm.

**Supplementary Table 1.** Structure parameters for $RuO_2$, $SrRuO_3$ and $Sr_2RuO_4$ based on the Reitveld refinement.

| | $RuO_2$ | $SrRuO_3$ | $Sr_2RuO_4$ |
|---|---|---|---|
| Crystal structure | *P*42/*mnm* | *Pnma* | *I*4/*mmm* |
| Lattice parameters (Å) | a=b=4.4965(3)<br>c=3.1035(2) | a=5.5738(4)<br>b=7.8514(6)<br>c=5.5385(4) | a=b=3.8769(3)<br>c=12.730(1) |
| Unit cell volume (Å$^3$) | 62.7481 | 242.3766 | 191.3364 |
| Bond length Ru-O (Å) | Ru−O1 ×4: 1.9852<br>Ru−O2 ×2: 1.9414 | Ru−O1 ×2: 1.9495<br>Ru−O1 ×2: 2.0077<br>Ru−O2 ×2: 2.0233 | Ru−O1 ×4: 1.9385<br>Ru−O2 ×2: 2.075 |
| Bond angle Ru-O-Ru (deg) | 128.5854<br>102.8291 | 151.9284<br>166.2429 | 180 |
| $R_p$ (%) | 5.37 | 5.38 | 6.65 |
| $R_{wp}$ (%) | 6.99 | 7.30 | 9.28 |
| $\chi 2$ | 1.202 | 3.965 | 6.424 |

**Supplementary Table 2.** Summary of HER activity in alkaline media (1 M KOH) for various representative catalysts, including Ru-based catalysts, metal oxides, metal/alloy, metal phosphides, metal sulfides, metal selenides, metal carbides, functional carbon and composites, etc.

| Catalysts | Substrate | Mass loading (mg cm$^{-2}$) | η @ 10 mA cm$^{-2}$ (mV) | Tafel slope (mV dec$^{-1}$) | References |
|---|---|---|---|---|---|
| **$Sr_2RuO_4$** | **Glass carbon** | **0.232** | **61** | **51** | **This work** |
| | | | **Ru-based catalysts** | | |
| $Ru@C_2N$ | N.A.[a] | 0.285 | 17 | 38 | *Nat. Nanotech.* **2017**, *12*, 441 |
| $Ru/C_3N_4/C$[b] | Glass carbon | 0.204 | 79 | N.A. | *J. Am. Chem. Soc.* **2016**, *138*, 16174 |
| $RuP_2@NPC$ | Glass carbon | 1 | 52 | 69 | *Angew. Chem. Int. Ed.* **2017**, *56*, 11559 |
| NiRu@NC | Glass carbon | 0.273 | 32 | 64 | *J. Mater. Chem. A* **2018**, *6*, 1376 |
| $Ni_{1.5}Co_{1.4}P@Ru$ | Glass carbon | 0.28 | 52 | 50 | *Chem. Commun.* **2017**, *53*, 13153 |
| $Cu_{2-x}S@Ru$ NPs | Glass carbon | 0.23 | 48 | 82 | *Small* **2017**, *13*, 1700052 |
| $Ru/MoS_2$ | Carbon paper | ~1 | 13 | 60 | *Nanoscale* **2017**, *9*, 16616 |
| 1D $RuO_2@CN_x$[c] | Glass carbon | ~0.212 | 95 | 70 | *ACS Appl. Mater. Interfaces* **2016**, *8*, 28678 |
| $RuO_2/Co_3O_4$ | Glass carbon | 0.285 | 89 | 91 | *RSC Adv.* **2017**, *7*, 3686 |
| | | | **Metal oxides** | | |
| 2-Cylce $NiFeO_x$ | Carbon fiber paper | 1.6 | 88 | 150 | *Nat. Commun.* ***2015***, *6*, 7261 |
| SNCF-NR | Glass carbon | 0.232 | 232 | 103 | *Adv. Energy Mater.* ***2017***, *7*, 1602122 |
| Pr0.5BSCF | Glass carbon | 0.232 | 237 | 45 | *Adv. Mater.* **2016**, *28*, 6442 |
| NiO NRs-m-Ov | Carbon fiber paper | 0.2 | 110 | 100 | *Nano Energy* **2018**, *43*, 103 |
| $NiCo_2O_4$ | Ni foam | ~1 | ~100 | 50 | *Angew. Chem. Int. Ed.* **2016**, *55*, 6290 |
| NiFe LDH[d] | Ni foam | N.A. | 240 | N.A. | *Science* **2014**, *345*, 1593 |
| S-CoO NRs | Carbon fiber paper | 0.486 | 73 | 82 | *Nat. Commun.* ***2017***, *8*, 1509 |
| *m*$MoO_3$[a] | Ni foam | 0.2 | 138 | 56 | *Adv. Energy Mater.* ***2016***, *6*, 1600528 |

| | | | | | |
|---|---|---|---|---|---|
| H- $MnMoO_4$ | Ni foam | 1.2 | ~220 | 36 | *J. Mater. Chem. A* ***2016***, *6*, 1600528 |
| **Metals/alloys** | | | | | |
| $Pt_3Ni_3$ nanowires | Glass carbon | N.A. | 50 | N.A. | *Angew. Chem. Int. Ed.* **2016**, *55*, 12859 |
| MnNi | Glass carbon | 0.28 | 360 | N.A. | *Adv. Funct. Mater.* **2015**, *25*, 393 |
| Co@NG | Glass carbon | 0.47 | 220 | 112 | *Adv. Funct. Mater.* **2016**, *26*, 4397 |
| NiMoN | Carbon cloth | ~1.1 | 109 | 95 | *Adv. Energy Mater.* ***2016***, *6*, 1600221 |
| Ni-BDT-A | Carbon cloth | 1 | 80 | 70 | *Chem* ***2017***, *3*, 122 |
| $CoN_x$/C | Glass carbon | 2 | 170 | 75 | *Nat. Commun.* ***2015***, *6*, 7992 |
| Mo-SAC[b] | Glass carbon | 0.408 | 132 | 90 | *Angew. Chem. Int. Ed.* **2017**, *56*, 16086 |
| Ni42-300 | N.A. | N.A. | 299 | ~117 | *Adv. Funct. Mater.* **2016**, *26*, 6402 |
| NiMo NWs | Ni foam | 0.41 | 270 | 86 | *Nano Energy* **2016**, *27*, 247 |
| Pd-$CN_x$[c] | Glass carbon | 0.215 | 122 | 150 | *ACS Catal.* ***2016***, *6*, 1929 |
| **Metal phosphides** | | | | | |
| MoP | Glass carbon | 0.86 | ~150 | 48 | *Energy Environ. Sci.* **2014**, *7*, 2624 |
| CoP | Carbon cloth | 0.92 | 209 | 129 | *J. Am. Chem. Soc.* **2014**, *136*, 7587 |
| . $Cu_{0.3}Co_{2.7}P$/NC | Glass carbon | 0.4 | 220 | 122 | *Adv. Energy Mater.* **2017**, *7*, 1601555 |
| Ni-P | Carbon paper | N.A. | 100 | ~85 | *Adv. Funct. Mater.* **2016**, *26*, 4067 |
| $Ni_5P_4$ | Ni foil | 25.8 | 150 | 53 | *Angew. Chem. Int. Ed.* **2015**, *127*, 12538 |
| Fe-CoP | Ti foil | 1.03 | 78 | 75 | *Adv. Mater.* **2017**, *29*, 1602441 |
| np-$(Co_{0.52}Fe_{0.48})_2P$ | N.A. | 2.5 | 79 | 40 | *Energy Environ. Sci.* **2016**, *9*, 2257 |
| Ce-doped CoP NWs | Ti plate | 0.2 | 92 | 64 | *Nano Energy* **2017**, *38*, 290 |
| CoMoP@C | Glass carbon | 0.354 | 81 | 56 | *Energy Environ. Sci.* **2017**, *10*, 788 |

| | | | | | |
|---|---|---|---|---|---|
| CoP-MNA | Ni foam | 6.2 | 54 | 51 | *Adv. Funct. Mater.* **2015**, *25*, 7337 |
| $Zn_xCo_{1-x}P$ | Titanium mesh | 1.52 | 67 | N.A. | *Adv. Energy Mater.* **2017**, *7*, 1700020 |
| NiCoP/rGO | Carbon fiber paper | 0.15 | 209 | 124 | *Adv. Funct. Mater.* **2016**, *26*, 6785 |
| | | | **Metal sulfides** | | |
| $Ni_3S_2$ | Ni foam | 1.6 | 223 | N.A. | *J. Am. Chem. Soc.* **2015**, *137*, 14023 |
| a-$Ni_3S_2$@NPC | Cu film | N.A. | 61 | 68 | *Nano Energy* **2017**, *36*, 85 |
| $Ni_{0.33}Co_{0.67}S_2$ nanowire | Ti foil | 0.3 | 88 | 118 | *Adv. Energy Mater.* **2015**, *5*, 1402031 |
| $Zn_{0.30}Co_{2.70}S_4$ | Glass carbon | 0.285 | 85 | N.A. | *J. Am. Chem. Soc.* **2016**, *138*, 1359 |
| Cu NDs/$Ni_3S_2$ NTs | Carbon fibers | 0.52 | 128 | 76 | *J. Am. Chem. Soc.* **2018**, *140*, 610 |
| $NiCo_2S_4$ NW | Ni foam | N.A. | 210 | 59 | *Adv. Funct. Mater.* **2016**, *26*, 4661 |
| Ni-doped $MoS_2$ | N.A. | 0.89 | 98 | 60 | *Energy Environ. Sci.* **2016**, *9*, 2789 |
| $MoS_2/Ni_3S_2$ | Ni foam | 9.7 | 110 | 83 | *Angew. Chem. Int. Ed.* **2016**, *55*, 6702 |
| | | | **Metal selenides** | | |
| $Ni_{0.89}Co_{0.11}Se_2$ MNSN | Ni foam | 2.62 | 85 | 52 | *Adv. Mater.* **2017**, *29*, 1606521. |
| NiSe | Ni foam | 2.8 | 96 | 120 | *Angew. Chem. Int. Ed.* **2015**, *54*, 9351 |
| c-$CoSe_2$ | Carbon cloth | 0.5 | 200 | 85 | *Adv. Mater.* **2016**, *28*, 7527 |
| | | | **Metal carbides** | | |
| $Mo_2C$ | Carbon paste | 0.8 | ~190 | 54 | *Angew. Chem. Int. Ed.* **2012**, *51*, 12703 |
| $MoC_x$ nano-octahedrons | Glass carbon | 0.8 | 151 | 59 | *Nat. Commun.* ***2015***, *6*, 6512 |
| $Mo_2C$/N-doped C | Glass carbon | 0.28 | 100 | 65 | *ACS Nano* **2016**, *10*, 11337 |
| N,P-doped $Mo_2C$@carbon nanospheres | Glass carbon | 0.9 | 50 | 71 | *ACS Nano* **2016**, *10*, 8851 |
| $Mo_2C$/CLCN | Glass carbon | 0.357 | ~200 | 55 | *Nano Energy* **2017**, *41*, 749 |

| | | | | | |
|---|---|---|---|---|---|
| **Functional carbon materials** | | | | | |
| C3N4@NG | Glass carbon | 0.1 | >600 | N.A. | *Nat. Commun.* **2014**, *5*, 3783 |
| N,S-CN | Glass carbon | N.A. | 380 | 103 | *ACS Nano* **2017**, *11*, 7293 |
| ONPPGC | Carbon cloth | 0.1 | 446 | 154 | *Energy Environ. Sci*. **2016**, *9*, 1210 |
| **Composite materials** | | | | | |
| Pt@2D-$Ni(OH)_2$[b] | Glass carbon | N.A. | 180 | 72 | *Nano Energy* **2017**, *31*, 456 |
| $Co(OH)_2$/PANI | Ni foam | N.A. | 90 | 92 | *Adv. Mater.* **2015**, *27*, 7051 |
| $CoO_x$@CN | Glass carbon | 0.12 | 232 | N.A. | *J. Am. Chem. Soc.* **2015**, *137*, 2688 |
| Co-NRCNTs | Glass carbon | 0.28 | 370 | N.A. | *Angew. Chem., Int. Ed.* **2014**, *53*, 4372 |
| Ni/NiO-CNT | Glass carbon | 0.28 | ~80 | 82 | *Nat. Commun.* **2014**, *5*, 4695 |
| Co/$Co_3O_4$ core/shell nanosheets | Ni foam | 0.85 | 95 | 44 | *Nano Lett.*, **2015**, *15*, 6015 |
| Co-C-N | Carbon paper | N.A. | 178 | 102 | *J. Am. Chem. Soc.* **2015**, *137*, 15070 |
| $Ni(OH)_2$/$MoS_2$ | Carbon cloth | N.A. | 80 | 60 | *Nano Energy* **2017**, *37*, 74 |
| Ni-NiO/N-rGO | Glass carbon | 0.21 | 260 | 67 | *Adv. Funct. Mater.* **2015**, *25*, 5799 |
| $TiO_2$ NDs/Co NSNTs | Carbon fibers | 0.75 | 108 | 62 | *Angew. Chem. Int. Ed.* **2017**, *56*, 2960 |
| Ni/NiS | Ni foam | N.A. | 230 | ~123 | *Adv. Funct. Mater.* **2016**, *26*, 3314 |
| $Co_{0.85}$Se/NiFe-LDH | Exfoliated graphene (EG) foil | 4 | 260 | 160 | *Energy Environ. Sci.* **2016**, 9, 478 |
| NiFe/$NiCo_2O_4$ | Ni foam | / | 105 | 88 | *Adv. Funct. Mater.* **2016**, *26*, 3515 |

[a]: N. A.=Not available.
[b]: The electrolyte is 0.1 M KOH.
[c]: The electrolyte is 0.5 M KOH.
[d]: The electrolyte is 1 M NaOH.

**Supplementary Table 3.** TOF values of $Sr_2RuO_4$ and some well-known HER catalysts in literatures.

| Catalysts | Electrolyte | TOF ($H_2$ $s^{-1}$) (η=0.1 V) | References |
|---|---|---|---|
| **$Sr_2RuO_4$** | **1 M KOH** | **0.90** | **This work** |
| Ru/NG | 1 M KOH | 0.35 | *ACS Appl. Mater. Interfaces*, 2017,9, 3785 |
| $Ni_5P_4$ | 1 M NaOH | 0.79 | *Energy & Environ. Sci.*, 2015, 8, 1027 |
| $NiCo_2P_x$ | 1 M KOH | 0.056 | *Adv. Mater.*, 2017, 29, 1605502 |
| Ni-Mo alloy | 2 M KOH | 0.05 | *ACS Catal.*, 2013, 3, 166 |
| Ni-$MoS_2$ | 1 M KOH | 0.08 | *Energy & Environ. Sci.*, 2016, 9, 2789 |
| S-CoO NRs | 1 M KOH | 0.41 | *Nat. Commun.*, 2017, 8, 1509 |
| $NiP_2$-$CeO_2$ | 1 M KOH | 0.593 | *Inorg. Chem.*, 2018, 57, 548 |
| $Mo_1N_1C_2$ | 0.1 M KOH | 0.465 | *Angew. Chem. Int. Ed.*,2017, 56, 16086 |
| $MoNi_4/MoO_2$@Ni | 1 M KOH | 0.4[a] | *Nat. Commun.*, 2017, 8, 15437 |

[a ]The overpotential is at 0.05 V.

**Supplementary Table 4.** Comprehensive comparison of HER activity among $RuO_2$, $SrRuO_3$ and $Sr_2RuO_4$.

| Catalysts | Wt.% (Ru) | η @ 10 mA $cm^{-2}$ (mV) | Tafel slope (mV $dec^{-1}$) | $j_0$ (mA $cm^{-2}$) | Specific activity @η=0.1V (mA $cm^{-2}_{BET}$) | TOF@η=0.1V ($s^{-1}$) |
|---|---|---|---|---|---|---|
| $RuO_2$ | 76.0 | 95 | 49 | 0.115 | 0.53 | 0.11 |
| $SrRuO_3$ | 42.7 | 101 | 67 | 0.409 | 2.005 | 0.35 |
| $Sr_2RuO_4$ | 29.7 | 61 | 51 | 0.898 | 7.93 | 0.90 |

**Supplementary Table 5.** Price of different noble metals[a]

| Metal | Symbol | Unit of Measure | U.S. |
|---|---|---|---|
| Platinum | Pt | troy ounce | $974.00 |
| Palladium | Pd | troy ounce | $1095.00 |
| Rhodium | Rh | troy ounce | $1700.00 |
| Iridium | Ir | troy ounce | $990.00 |
| Ruthenium | Ru | troy ounce | $195.00 |
| Osmium | Os | troy ounce | $400.00 |
| Rhenium | Re | pound | $1290.00 |
| Gold | Au | troy ounce | $1322.89 |

[a]The prices for various noble metals are from the BASF corporation website on January 08, 2018. (https://apps.catalysts.basf.com/apps/eibprices/mp/)

**Supplementary Table 6**. The adsorption free energy of H* ($\Delta G_{H*}$) on $Sr_2RuO_4$, $SrRuO_3$ and $RuO_2$ surfaces. Adsorption energies were calculated in vacuum and with solvation effect considered.

| Site | Vacuum (eV) | Solvent (eV) |
|---|---|---|
| O @ $Sr_2RuO_4$ | -0.08 | -0.10 |
| Ru @ $SrRuO_3$ | -0.23 | -0.26 |
| O @ $SrRuO_3$ | -0.47 | -0.44 |
| Ru @ $RuO_2$ | 0.48 | 0.55 |
| $O^{bri}$ @ $RuO_2$ | -0.80 | -0.79 |
| O @ $RuO_2$ | 0.36 | 0.23 |

**Supplementary Table 7.** O 1s XPS peak deconvolution results.

| Electrocatalysts | lattice $O^{2-}$ | $O_2^{2-}/O^-$ | -OH | $H_2O$ |
|---|---|---|---|---|
| $Sr_2RuO_4$ | 16.1% | 20.4% | 57.0% | 6.5% |
| $SrRuO_3$ | 38.1% | 31.2% | 22.1% | 8.6% |
| $RuO_2$ | 52.6% | 22.5% | 15.0% | 9.9% |

O 1s XPS spectra can be deconvoluted into four different characteristic peaks, i.e., lattice oxygen species (~528.8 eV for $O^{2-}$), highly oxidative oxygen species (~530.1 eV for $O_2^{2-}/O^-$), hydroxyl groups or the surface adsorbed oxygen (~531.1 eV for -OH), and adsorbed molecular water or carbonates (~532.4 eV for $H_2O$)[8-13].

## Supplementary Note 1. Turnover frequency calculations

In this study, TOF values were calculated according to previous reported method[14-21] with detailed process shown as follows:

(1) To calculate the per-site turnover frequency (TOF), we used the following formula:

$$\mathrm{TOF}=\frac{\#\text{number of total hydrogen } turnovers/\mathrm{cm}^2_{\mathrm{geo}}}{\#\text{number of active sites}/\mathrm{cm}^2_{\mathrm{geo}}} \quad (1)$$

The total number of hydrogen turnovers was calculated from the current density according to:

$$\text{No. of } H_2 = \left(j\frac{\mathrm{mA}}{\mathrm{cm}^2_{\mathrm{geo}}}\right)\left(\frac{1\ \mathrm{C\ s^{-1}}}{1000\ \mathrm{mA}}\right)\left(\frac{1\ \mathrm{mol\ e^{-1}}}{96485.3\ \mathrm{C}}\right)\left(\frac{1\ \mathrm{mol\ H_2}}{2\ \mathrm{mol\ e^-}}\right)\left(\frac{6.022\times10^{23}\ \mathrm{H_2\ molecules}}{1\ \mathrm{mol\ H_2}}\right)=3.12\times10^{15}\ \frac{\mathrm{H_2/s}}{\mathrm{cm}^2_{\mathrm{geo}}}\ \text{per}\frac{\mathrm{mA}}{\mathrm{cm}^2_{\mathrm{geo}}}$$

(2) We estimate the number of active sites as the number of surface active oxygen sites (as to be confirmed by the following calculations with H* more easily adsorbing in O than Ru) from the unit cell of the $RuO_2$, $SrRuO_3$ and $Sr_2RuO_4$ crystal structure.

The active sites per real surface area are calculated from the following formula:

$$\text{No. of active sites} = \left(\frac{\text{No.of atoms/unit cell}}{\text{Volume/unit cell}}\right)^{\frac{2}{3}} \quad (2)$$

From Figure S10 we can calculate the number of active sites per real surface area for $RuO_2$, $SrRuO_3$ and $Sr_2RuO_4$:

$$\text{No. of active sites } (RuO_2) = \left(\frac{4\ \text{atoms/unit cell}}{62.75\ \text{Å}^3\ \text{/unit cell}}\right)^{\frac{2}{3}} = 1.595\times10^{15}\ \text{atoms cm}^{-2}_{\text{real}}$$

$$\text{No. of active sites } (SrRuO_3) = \left(\frac{12\ \text{atoms/unit cell}}{242.38\ \text{Å}^3\ \text{/unit cell}}\right)^{\frac{2}{3}} = 1.348\times10^{15}\ \text{atoms cm}^{-2}_{\text{real}}$$

$$\text{No. of active sites } (Sr_2RuO_4) = \left(\frac{4\ \text{atoms/unit cell}}{191.34\ \text{Å}^3\ \text{/unit cell}}\right)^{\frac{2}{3}} = 7.59\times10^{14}\ \text{atoms cm}^{-2}_{\text{real}}$$

(3) The real surface area for HER is calculated from the electrochemical active surface area (ECSA), which can be converted from the specific capacitance. The specific capacitance for a flat surface is generally found to be in the range of 20-60 μF $cm^2_{geo}$. In the following calculations of TOF we assume an average of 40 μF $cm^2_{geo}$ for $RuO_2$, $SrRuO_3$ and $Sr_2RuO_4$.

$$A_{\mathrm{ECSA}} = \frac{\text{specific capacitance}}{40\ \mu\mathrm{F\ cm}^2_{\mathrm{geo}}\ \text{per cm}^2_{\mathrm{ECSA}}} \quad (3)$$

From Figure S8 we can calculate ECSA for $RuO_2$, $SrRuO_3$ and $Sr_2RuO_4$:

$$A_{\mathrm{ECSA}}(RuO_2)= \frac{1.82\ \mathrm{mF}/0.196\ \mathrm{cm}^2_{\mathrm{geo}}}{40\ \mu\mathrm{F\ cm}^2_{\mathrm{geo}}\ \text{per cm}^2_{\mathrm{ECSA}}} =232.3\ \mathrm{cm}^2_{\mathrm{ECSA}}$$

$$A_{\mathrm{ECSA}}(SrRuO_3)= \frac{0.50\ \mathrm{mF}/0.196\ \mathrm{cm}^2_{\mathrm{geo}}}{40\ \mu\mathrm{F\ cm}^2_{\mathrm{geo}}\ \text{per cm}^2_{\mathrm{ECSA}}} =63.8\ \mathrm{cm}^2_{\mathrm{ECSA}}$$

$$A_{\mathrm{ECSA}}(Sr_2RuO_4)= \frac{0.92\ \mathrm{mF}/0.196\ \mathrm{cm}^2_{\mathrm{geo}}}{40\ \mu\mathrm{F\ cm}^2_{\mathrm{geo}}\ \text{per cm}^2_{\mathrm{ECSA}}} =117.3\ \mathrm{cm}^2_{\mathrm{ECSA}}$$

(4) Finally, the plot of current density can be converted into a TOF plot according to the following formula:

$$\mathrm{TOF}=\frac{\left(3.12\times10^{15}\ \frac{\mathrm{H2/s}}{\mathrm{cm}^2_{\mathrm{geo}}}\ \text{per}\frac{\mathrm{mA}}{\mathrm{cm}^2_{\mathrm{geo}}}\right)\times|j|}{\text{No.of active sites}\times A_{\mathrm{ECSA}}} \quad (4)$$

## Supplementary Note 2. Structural details in DFT calculations

Surfaces were modelled using slabs exposing the (001) and (010) planes of $Sr_2RuO_4$ and $SrRuO_3$, respectively. For $Sr_2RuO_4$ we considered the SrO termination as this is the most relevant plane demonstrated by advanced imaging techniques before. For $SrRuO_3$, we considered the $RuO_2$ termination given that this commonly the most relevant termination for $ABO_3$ systems. The slabs were symmetric consisting of 9-11 layers, which is sufficient to converge the adsorption energies to within 0.01 eV. We used a $2 \times 2$ cell for $Sr_2RuO_4$ and a $a\sqrt{2} \times a\sqrt{2}$ for the *Pnma* $SrRuO_3$ structure. The top 4 layers were relaxed while the bottom layers were fixed to their corresponding bulk positions. For $Ru_{hcp}$ we modelled the (0001) surface using a $3 \times 3$ 5 layers slab with the bottom two layers fixed. Pt(111) was modeled using a $\sqrt{3} \times \sqrt{3}$ reconstruction.

## Supplementary Note 3. Coverage Effect

Coverage effects were accounted for by calculating the energy needed to add one hydrogen at a time to the surface under consideration. This differential adsorption energy is defined as:

$$\Delta_{\mathrm{H}^*}^{\mathrm{diff}} = E(nH^*) - E[(n-1)H^*] - \frac{1}{2}E(H_2) \quad (5)$$

Where $E(nH^*)$ and $E[(n-1)H^*]$ are the DFT energies of the surface with $n$ and $n-1$ hydrogen intermediates, and $E(H_2)$ is the energy of a hydrogen molecule in the gas phase. The coverage is defined with respect to the active sites per supercell used. For SrO terminations in $Sr_2RuO_4$ there are four O active sites. Sr atoms are inactive for HER with $\Delta G_{H^*} = 2.45$ eV. The $RuO_2$ termination in $SrRuO_3$ is more complex as both Ru and O can act as possible active sites. There are 4 Ru and 8 O sites in our models. To simplify our models and reduce the number of isomeric configurations of H we study O and Ru sites separately. In reality however, H adsorption would be possible on both sites simultaneously.

## Supplementary References


[1] Liu, R. S., Jang, L.-Y., Hung, H.-H & Tallon, J. L. Determination of Ru valence from x-ray absorption near-edge structure in $RuSr_2GdCu_2O_8$-type superconductors. *Phys. Rev. B* **63**, 212507 (2001).

[2] Hu, Z. *et al.* Multiplet effects in the Ru $L_{2,3}$ x-ray-absorption spectra of Ru (IV) and Ru (V) compounds. *Phys. Rev. B* **61**, 5262 (2000).

[3] Agrestini S. *et al.* Electronically highly cubic conditions for Ru in α-$RuCl_3$. *Phys. Rev. B* **96**, 161107 (2017).

[4] Ferreira, P. J. *et al.* Instability of Pt/C electrocatalysts in proton exchange membrane fuel cells a mechanistic investigation. *J. Electrochem. Soc*. **152**, A2256-A2271 (2005).

[5] Suntivich, J., May, K. J., Gasteiger, H. A., Goodenough, J. B. & Shao-Horn, Y. A perovskite oxide optimized for oxygen evolution catalysis from molecular orbital principles. *Science* **334**, 1383-1385 (2011).

[6] Chen, X. *et al.* Oxygen evolution reaction on $La_{1-x}Sr_xCoO_3$ perovskites: a combined experimental and theoretical study of their structural, electronic, and electrochemical properties. *Chem. Mater.* **27**, 7662-7672 (2015).

[7] Crumlin, E. J. *et al.* Surface strontium enrichment on highly active perovskites for oxygen electrocatalysis in solid oxide fuel cells. *Energy Environ. Sci.* **5**, 6081-6088 (2012).

[8] Wang, Y. G. *et al.* Nanocasted synthesis of mesoporous $LaCoO_3$ perovskite with extremely high surface area and excellent activity in methane combustion, *J. Phys. Chem. C* **112** , 15293-15298 (2008).

[9] Liu, R., Liang, F., Zhou, W., Yang, Y. & Zhu, Z. Calcium-doped lanthanum nickelate layered perovskite and nickel oxide nano-hybrid for highly efficient water oxidation, *Nano Energy* **12**, 115-122 (2015).

[10] Zhu, Y. *et al.* Enhancing electrocatalytic activity of perovskite oxides by tuning cation deficiency for oxygen reduction and evolution reactions. *Chem. Mater*. **28**, 1691-1697 (2016).

[11] Liang, F., Yu, Y., Zhou, W., Xu, X. & Zhu, Z. Highly defective $CeO_2$ as a promoter for efficient and stable water oxidation. *J. Mater. Chem. A* **3**, 634-640 (2015).

[12] Xu, X. *et al.* A perovskite electrocatalyst for efficient hydrogen evolution reaction. *Adv. Mater.* **28**, 6442-6448 (2016).

[13] Zhou, W., Zhao, M., Liang, F., Smith, S. & Zhu, Z. High activity and durability of novel perovskite electrocatalysts for water oxidation. *Mater. Horiz.* **2**, 495-501 (2015).

[14] Kibsgaard, J. & Jaramillo, T. F. Molybdenum phosphosulfide: an active, acid-stable, earth-abundant catalyst for the hydrogen evolution reaction. *Angew. Chem. Int. Ed.* **53**, 14433-14437 (2014).

[15] Benck, J. D., Chen, Z., Kuritzky, L. Y., Forman, A. J. & Jaramillo, T. F. Amorphous molybdenum sulfide catalysts for electrochemical hydrogen production: insights into the origin of their catalytic activity. *ACS Catal.* **2**, 1916-1923 (2012).

[16] Benck, J. D., Hellstern, T. R., Kibsgaard, J., Chakthranont, P. & Jaramillo, T. F. Catalyzing the hydrogen evolution reaction (HER) with molybdenum sulfide nanomaterials. *ACS Catal.* **4**, 3957-3971 (2014).

[17] Wang, X. *et al.* Novel porous molybdenum tungsten phosphide hybrid nanosheets on

carbon cloth for efficient hydrogen evolution. *Energy Environ. Sci.* **9**, 1468-1475 (2016).

[18] Kibsgaard, J. *et al.* Designing an improved transition metal phosphide catalyst for hydrogen evolution using experimental and theoretical trends. *Energy Environ. Sci.* **8**, 3022-3029 (2015).

[19] Zhang, R. *et al.* Ternary $NiCo_2P_x$ nanowires as pH-universal electrocatalysts for highly efficient hydrogen evolution reaction. *Adv. Mater.* **29**, 1605502 (2017).

[20] Tang, C. *et al.* Sulfur-decorated molybdenum carbide catalysts for enhanced hydrogen evolution. *ACS Catal.* **5**, 6956-6963 (2015).

[21] Y. Zheng, *et al.* High electrocatalytic hydrogen evolution activity of an anomalous ruthenium catalyst. *J. Am. Chem. Soc.* **138**, 16174-16181 (2016).
